# Itraconazole inhibits nuclear delivery of extracellular vesicle cargo by disrupting the entry of late endosomes into the nucleoplasmic reticulum

**DOI:** 10.1002/jev2.12132

**Published:** 2021-08-14

**Authors:** Mark F. Santos, Germana Rappa, Jana Karbanová, Simona Fontana, Maria Antonietta Di Bella, Marshall R. Pope, Barbara Parrino, Stella Maria Cascioferro, Giulio Vistoli, Patrizia Diana, Girolamo Cirrincione, Goffredo O. Arena, Gyunghwi Woo, Kevin Huang, Tony Huynh, Marta Moschetti, Riccardo Alessandro, Denis Corbeil, Aurelio Lorico

**Affiliations:** ^1^ College of Medicine Touro University Nevada Henderson Nevada USA; ^2^ Biotechnology Centre and Centre for Molecular and Cellular Bioengineering Technische Universität Dresden Dresden Germany; ^3^ Department of Biomedicine, Neurosciences and Advanced Diagnostics University of Palermo Palermo Italy; ^4^ Proteomics Facility University of Iowa Iowa City Iowa USA; ^5^ Dipartimento di Scienze e Tecnologie Biologiche Chimiche e Farmaceutiche Università degli Studi di Palermo Palermo Italy; ^6^ Dipartimento di Scienze Farmaceutiche Università degli Studi di Milano Milan Italy; ^7^ Department of Surgery McGill University Montréal Québec Canada; ^8^ Fondazione Istituto G. Giglio Cefalù Italy; ^9^ Institute for Biomedical Research and Innovation (IRIB) National Research Council (CNR) Palermo Italy; ^10^ Mediterranean Institute of Oncology Viagrande Italy

**Keywords:** cancer, endosome, exosome, intercellular communication, metastasis, micro‐vesicle, nucleoplasmic reticulum

## Abstract

Extracellular vesicles (EVs) are mediators of intercellular communication under both healthy and pathological conditions, including the induction of pro‐metastatic traits, but it is not yet known how and where functional cargoes of EVs are delivered to their targets in host cell compartments. We have described that after endocytosis, EVs reach Rab7^+^ late endosomes and a fraction of these enter the nucleoplasmic reticulum and transport EV biomaterials to the host cell nucleoplasm. Their entry therein and docking to outer nuclear membrane occur through a tripartite complex formed by the proteins VAP‐A, ORP3 and Rab7 (VOR complex). Here, we report that the antifungal compound itraconazole (ICZ), but not its main metabolite hydroxy‐ICZ or ketoconazole, disrupts the binding of Rab7 to ORP3–VAP‐A complexes, leading to inhibition of EV‐mediated pro‐metastatic morphological changes including cell migration behaviour of colon cancer cells. With novel, smaller chemical drugs, inhibition of the VOR complex was maintained, although the ICZ moieties responsible for antifungal activity and interference with intracellular cholesterol distribution were removed. Knowing that cancer cells hijack their microenvironment and that EVs derived from them determine the pre‐metastatic niche, small‐sized inhibitors of nuclear transfer of EV cargo into host cells could find cancer therapeutic applications, particularly in combination with direct targeting of cancer cells.

## 1 INTRODUCTION

Extracellular vesicles (EVs), including exosomes, microvesicles and other types of membrane particles act as mediators of intercellular communication in tissues and organs under healthy and pathological conditions (Maas et al., 2017; Mathieu et al., 2019; Raposo & Stoorvogel, 2013). Their role in modulation of immune responses, cell differentiation, and epithelial‐mesenchymal transition has been demonstrated. In cancer, their dysregulated secretion and altered cargoes (i.e., proteins, lipids and nucleic acids) can promote tumour growth and lead to metastasis (Abdouh et al., 2017; Hoshino et al., 2015; Peinado et al., 2012), suggesting that targeting EVs may prevent metastasis. However, our knowledge about EV cargos’ subcellular/molecular sites of action in recipient cells is still fragmented. This is specifically true for proteins and nucleic acids associated with EVs that shuttle to the nucleus of host cells (Santos et al., 2018).

Upon the cellular internalization of EVs by various endocytosis mechanisms (reviewed in Ref. [Corbeil et al., 2020]), the fusion of endocytosed EVs with late endosome membrane and/or the breakdown of the latter could result in exposure of EVs and/or their cargo to cellular cytosol (Joshi et al., 2020). The transfer of EV contents through endosomal compartment to the endoplasmic reticulum (ER) has been proposed (Heusermann et al., 2016). Recently, we described a novel intracellular pathway whereby EV‐containing late endosomes enter in the nucleoplasmic reticulum through type II nuclear envelope invaginations (NEI) and transfer EV cargoes into the nucleoplasm of recipient cells (Rappa et al., 2017). A tripartite protein complex named VOR, containing ER‐associated, vesicle‐associated membrane protein (VAMP)‐associated protein A (**V**AP‐A), oxysterol‐binding protein (OSBP)‐related protein‐3 (**O**RP3) and late endosome‐associated small GTPase **R**ab7 orchestrates entry and/or docking of EV‐containing late endosomes into NEI (Santos et al., 2018). Silencing VAP‐A or ORP3 abrogated the localization of Rab7^+^ late endosomes in NEI, and the subsequent nuclear transport of endocytosed EV‐derived components (Santos et al., 2018).

One VOR complex protein, namely ORP3, drew our attention as a potential therapeutic target because other members of the OSBP family, regulate the lipid exchange, notably sterols found at membrane contact sites between organelles (Eden, 2016; Olkkonen, 2015; Weber‐Boyvat et al., 2015). OSBP proteins contain a highly conserved C‐terminal OSBP‐related domain (ORD), whose hydrophobic pocket binds a single sterol, notably cholesterol and 25‐hydroxycholesterol (25‐HC) (Raychaudhuri & Prinz, 2010; Suchanek et al., 2007). OSBP‐related proteins are involved in diverse functions ranging from lipid sensing and trafficking to sterol homeostasis and cell signalling. ORP3 and VAP‐A, an integral type II membrane protein, reportedly establish a contact zone between ER and plasma membrane and regulate the activity of GTPase R‐Ras (Lehto et al., 2005; Olkkonen, 2015; Weber‐Boyvat et al., 2015). The interaction of ORP3 and VAP‐A occurs via two specific sequence motifs formed by two phenylalanines in an acidic tract (FFAT) (Loewen et al., 2003; Olkkonen, 2015; Weber‐Boyvat et al., 2015). A pleckstrin homology (PH) domain mediates ORP3 interaction with phosphoinositides in non‐ER organelles (Olkkonen, 2015), potentially including late endosomes (Santos et al., 2018). Unlike the OSBP‐related protein 1L (ORP1L), ORP3 does not contain an ankyrin repeat domain that mediates the Rab7 interaction at the contact zone of the ER‐late endosome (Olkkonen, 2015; Rocha et al., 2009). The question of whether the R‐Ras‐binding site of ORP3 is involved in the Rab7 interaction is open.

In search for drugs that could inhibit ORP3 function as component of the VOR complex, we came across ORPphilin molecules, such as anti‐proliferative natural product OSW‐1 (Burgett et al., 2011) and the FDA‐approved antifungal azole itraconazole (ICZ). Through the inhibition of lanosterol 14α‐demethylase, ICZ has clinically efficacious antifungal activity (Georgopapadakou & Walsh, 1996). Also, ICZ has been shown to interfere with intracellular trafficking of cholesterol, resulting in abnormal accumulation of cholesterol in the endosomal/lysosomal compartments (Liu et al., 2014; Trinh et al., 2017; Xu et al., 2010). An inhibitory activity of ICZ on enterovirus and hepatitis C virus replication has been attributed to OSBP and ORP4 inhibition (Strating et al., 2015). Its five ring‐backbone structure and the sec‐butyl chain are important for antiviral activity, whereas the triazole moiety, which is critical for the antifungal activity (Bauer et al., 2018), is not. For cancer treatment, ICZ has been repurposed because of its effect on the Hedgehog signalling pathway and angiogenesis (Pounds et al., 2017).

Here, we report that ICZ disrupts the VOR complex and inhibits EV‐mediated pro‐metastatic morphological transformation and migratory properties of colon cancer cells. These effects do not require the ICZ moieties responsible for antifungal activity or interference with intracellular cholesterol distribution, suggesting a novel mechanism of ICZ action. Altogether, we provide evidence that the VOR complex (and its pathway) is a novel drug target to impair the intercellular communication in the cancer microenvironment, and hence prevent metastases.

## 2 MATERIALS AND METHODS

### 2.1 Chemicals

ICZ (cis‐4[4‐4‐4[[2‐(2‐4‐dichlorophenyl)‐2‐(1H‐1,2,4,triazol‐1‐methyl)‐1,3‐dioxolan‐4‐yl]‐1‐piperazinyl]phenyl]‐2,4‐dihydro‐2‐(1‐methyl‐propyl)‐3H‐1,2,4‐triazol‐3‐one; MW: 705.64) and ketoconazole were purchased from Sigma‐Aldrich (catalogue numbers #I6657 and #K1003, respectively, St‐Louis, MO, USA), while OSW‐1 ((3β,16β)‐3,17‐dihydroxy‐22‐oxocholest‐5‐en‐16‐yl‐2‐O‐acetyl‐3‐O‐[2‐O‐(4‐methoxybenzoyl)‐β‐D‐xylopyranosyl]‐α‐L‐arabinopyranoside) was from Alfa Chemistry (#ACM145075816, Ronkonkoma, NY, USA). The hydroxy‐ICZ (H‐ICZ), thapsigargin, imipramine and 25‐HC were all purchased from Cayman Chemical (#22576, #10522, #15890 and #11097, respectively, Ann Arbor, MI, USA). Stock solutions were prepared at 10 mM in dimethyl sulfoxide (DMSO) (VWR International, Radnor, PA, USA) for ICZ, H‐ICZ, ketoconazole and thapsigargin; 100 mM for imipramine; 20 mM for 25‐HC; and 1 mM for OSW‐1. Filipin complex from *Streptomyces filipinensis* was obtained from Sigma‐Aldrich (#F9765), and the stock solution prepared at 25 mg/ml in DMSO. λ‐phosphatase was also purchased from Sigma‐Aldrich (#P9614) at a stock solution of 400,000 units (U) per ml.

### 2.2 Synthesis of PRR846 and PRR851 compounds

All materials and solvents were purchased from commercial sources and used without further purification. All melting points were obtained on a Büchi‐Tottoly capillary apparatus (Büchi, Cornaredo, Italy) and have not been corrected. IR spectra were determined in bromoform with a Shimadzu FT/IR 8400S spectrophotometer (Shimadzu Corporation, Milan, Italy). ^1^H spectra were measured at 50.0 MHz in DMSO‐d6 solution, using a Bruker Avance II series 200 MHz spectrometer (Bruker, Milan, Italy). Column chromatography was performed with Merck silica gel 230–400 mesh ASTM or with a Büchi Sepacor chromatography module (prepacked cartridge system). Elemental analyses (C, H, N) were within ± 0.4% of theoretical values and were performed with a VARIO EL III elemental analyser (Elementar, Langenselbold, Germany). Purity of all the tested compounds was greater than 95%, determined by HPLC (Agilent 1100 Series).

For the synthesis of 4‐(4‐chlorophenyl)‐2,4‐dihydro‐3H‐1,2,4‐triazol‐3‐one (PRR846), triethyl orthoformate (17.51 mmol, 2.9 ml), *p*‐toluene sulphonic acid (1.5 mmol, 295 mg) and methyl carbazate (17.51 mmol, 1.6 g), were added to a solution of 4‐chloroaniline (11.75 mmol, 1.5 g) in 100 ml methanol under nitrogen. The resulting reaction mixture was heated under reflux for 3 h. Once cooled at 20°C a solution of sodium methoxide in methanol, prepared by addition of sodium (17.51 mmol, 403 mg) to methanol (30 ml), was added to the mixture that was heated under reflux for two additional hours. The solvent was removed at reduced pressure and crushed ice was added to the reaction crude material. The resulting white precipitate was filtered off and recrystallized from methanol to afford the pure compound. Yield: 99%, white solid; mp: 101–102°C; IR: 3122 (NH), 1714 (CO) cm^−1^; ^1^H NMR (200 MHz, DMSO‐ *d*_6_) δ: 7.55‐7.61 (2H, m, ArH), 7.72‐7.79 (2H, m, ArH), 8.42 (1H, s, H‐3), 12.02 (1H, s, NH); anal. calculated for C_8_H_6_ClN_3_O (MW: 195.61): C, 49.12; H, 3.09; N, 21.48%. Found: C, 49.36; H, 2.95; N, 21.60%.

The synthesis of 2‐(butan‐2‐yl)‐4‐(4‐chlorophenyl)‐2,4‐dihydro‐3H‐1,2,4‐triazol‐3‐one (PRR851) was performed from the compound PRR846. To a PRR846 solution (4.1 mmol, 0.8 g) in DMSO (10 ml), Na_2_CO_3_ (8.2 mmol, 869 mg), 18‐crown‐6 (4.1 mmol, 1.08 g) and 2‐bromobutane (5.33 mmol, 0.6 ml) were added. The reaction mixture was heated at 60°C for 8 h. Once cooled, distilled water was added and the mixture extracted with ethyl acetate (3 × 100 ml). The organic layers were dried over anhydrous Na_2_SO_4_, filtered and evaporated *in vacuo*. The crude material was purified by silica gel column chromatography using dichloromethane: ethyl acetate, 9:1 as eluent. Yield: 80%, white solid; mp: 102–103°C; IR: 1689 (CO) cm^−1^; ^1^H NMR (200 MHz, DMSO‐ *d*_6_) δ: 0.79 (3H, t, *J =* 7.4 Hz, CH_3_), 1.29 (3H, d, *J =* 6.7 Hz, CH_3_), 1.61‐1.81 (2H, m, CH_2_), 4.08‐4.18 (1H, m, CH), 7.54‐7.60 (2H, m, ArH), 7.74‐7.86 (2H, m, ArH), 8.51 (1H, s, H‐3); anal. calculated for C_12_H_14_ClN_3_O (MW: 251.71): C, 57.26; H, 5.61; N, 16.69%. Found: C, 57.01; H, 5.81; N, 16.48%.

### 2.3 Synthesis of fluorescent PRR898 compound

The synthesis of 2‐ethyl‐4‐[4‐(7‐nitro‐benzo[1,2,5]oxadiazol‐4‐ylamino)‐phenyl]‐2,4‐dihydro‐[1,2,4]triazol‐3‐one (PRR898) was performed from the compound 4‐(4‐amino‐phenyl)‐2‐ethyl‐2,4‐dihydro‐[1,2,4]triazol‐3‐one in which the fluorescent 7‐nitrobenzofurazan group was linked, employing the same synthetic procedure as previously reported (Barresi et al., 2018).

### 2.4 Molecular modelling

The primary sequence of the human ORP3 protein was retrieved from Uniprot (entry: Q9H4L5, OSBL3_HUMAN) and was submitted to the online Swiss‐Model server to generate the corresponding homology model. Among the proposed possible templates, the ORP3 protein was modelled by using the recently resolved ORP1 in complex with cholesterol (PDB Id: 5ZM5) (Dong et al., 2019) chosen due to its sequence identity equal to 43.4% and the coverage, which allows the oxysterol binding domain of ORP3 to be completely modelled (Lehto et al., 2001). The generated model was then completed by adding the missing hydrogen atoms, and the structure underwent energy minimization using nanoscale molecular dynamics Namd2 software (Acun et al., 2018) by keeping fixed the backbone atoms to retain the predicted folding. The structure of the simulated ligands was optimized by the PM7 semi‐empirical method (Stewart, 2013). The ORP3 binding cavity was defined by superimposing the homology model with the experimental template. In this way, the corresponding position of the cholesterol within the ORP3 model was derived, thus allowing a precise definition of the binding pocket to be used in the following docking simulations. In detail, docking simulations were performed using the docking software PLANTS (Korb et al., 2009), focusing the search within an 8 Å radius sphere around the so derived cholesterol position; for each ligand/drug 10 possible configurations were generated and ranked by the ChemPLP scoring function with speed equal to one. The generated complexes were finally minimized by keeping fixed the atoms outside an 8 Å radius sphere around the bound ligand and rescored by using ReScore^+^ tools (Vistoli et al., 2017).

The multiple sequence alignment of the ORD domain for seven ORP isoforms plus human OSBP was computed using Clustal X (Larkin et al., 2007). Emphasis was placed on the main residues involved in PRR851 binding.

### 2.5 Antibodies

The primary and secondary antibodies (Ab) used in this study, their working concentrations and validation are described in Tables S1 and S2, respectively.

### 2.6 Cell culture

All cells used in this study and their validation are described in Table S3. Human HeLa (ATCC^®^CCL‐2^TM^), fibroblast BJ (ATCC^®^CRL‐2522^TM^), SW480 (CCL‐228^TM^) and SW620 cells (CCL‐227^TM^) were obtained from the American Type Culture Collection (ATCC, Manassas, VA, USA). FEMX‐I cells were originally derived from a lymph node metastasis of a patient with malignant melanoma and found to be highly metastatic in immunodeficient mice (Fodstad et al., 1988; Rappa et al., 2008) and wild type for BRAF, PTEN and NRAS (Rappa et al., 2017). FEMX‐I cells were authenticated by morphology and proteomics (Rappa et al., 2013a). FEMX‐I, SW480 and SW620 cells were cultured in RPMI‐1640 medium (#10‐041‐CV, Corning Inc., NY, USA), while HeLa and fibroblast BJ cells were kept in DMEM (#11995065, Thermo Fisher Scientific, Waltham, MA, USA). Human primary bone marrow‐derived mesenchymal stromal cells (MSCs) were originally acquired from D. J. Prockop (Texas A&M University) and prepared under a protocol approved by the Texas A&M Institutional Review Board. Cells were cultured in MEMα (#10‐022‐CV, Corning Inc.) and were used between passages three and five (Larson et al., 2008). All cell culture media were supplemented with 10% fetal bovine serum (FBS, #26140079), 2 mM L‐glutamine (#25030081), 100 U/ml penicillin and 100 μg/ml streptomycin (#15140122), all from Thermo Fisher Scientific. Cells were incubated at 37°C in a 5% CO_2_ humidified incubator. Cells were regularly verified for absence of mycoplasma contamination by either their staining with 4′,6‐diamidino‐2‐phenylindole (DAPI; #D9542, Sigma‐Aldrich) and visualization under a fluorescent microscope or polymerase chain reaction using the MycoSEQ Mycoplasma Detection Kit (#4460626, Thermo Fisher Scientific), according to the manufacturer's protocol.

### 2.7 Plasmids

C‐terminal green fluorescent protein (GFP)‐tagged fusion protein versions of CD9 (RG202000; OriGene Technologies, Rockville, MD, USA) or VAP‐A (HG11412‐ACG; Sino Biological, Beijing, China) were expressed in vectors pCMV6‐AC‐GFP or pCMV3‐C‐GFPSpark, which contain neomycin and hygromycin resistance genes, respectively. For gene knockdown, shRNA plasmids targeting VAP‐A (HSH022333‐nH1; Accession No. NM_003574.5), VAP‐B (HSH022331‐nH1; NM_004738.3), and ORP3 (HSH006982‐nH1; NM_015550.2) were purchased from GeneCopoeia (Rockville, MD, USA). A scrambled shRNA plasmid (CSHCTR001‐nH1; GeneCopoeia) was used as control. These plasmids contain the puromycin resistance gene. For each protein of interest, a pool of target sequences was utilized after their individual evaluation, as described previously (Santos et al., 2018). Two distinct shRNA plasmids were selected for VAP‐A (5′‐ CCACACAGTGTTTCACTTAAT‐3′ and 5′‐ GCACATTGAGTCCTTTATGAA‐3′) and VAP‐B (5′‐ GGATGACACCGAAGTTAAGAA‐3′ and 5′‐ GGTAAATTGGATTGGTGGATC‐3′), whereas four shRNA plasmids were selected for ORP3 (5′‐ CCATGTTTCCACATGAAGTTA‐3′, 5′‐CCTCCAATCCTAATTTGTCAA‐3′, 5′‐ GCCCATAAAGTTTACTTCACT‐3′, and 5′‐ GGAGAAACATATGAATGTATT‐3′).

### 2.8 Transfection

FEMX‐I cells were transfected with CD9‐GFP plasmid (500 ng) using FuGeneHD Transfection Reagent (#E2311, Promega, Madison, WI, USA) or VAP‐A‐GFP plasmid using Lipofectamine 3000 (#L3000008, Thermo Fisher Scientific). Transfected cells were selected by introducing 400 μg/ml of Geneticin (G418 Sulphate, #10131027, Thermo Fisher Scientific) or 200 μg/ml of hygromycin (#H3274, Sigma‐Aldrich) into the culture medium. To inhibit VAP‐A, VAP‐B, or ORP3 expression, SW480 cells were transfected with pooled shRNA plasmids (500 ng) using lipofectamine 3000. After transfection, stable cell lines were selected by 1 μg/ml puromycin (#P9620, Sigma‐Aldrich) for 7 days. All selection antibiotics were removed from the medium at least 1 week before experiments.

### 2.9 Baculovirus‐based expression

The baculovirus‐based BacMam 2.0 CellLight Late Endosomes‐red fluorescent protein (RFP) fusion protein and ER‐GFP or ‐RFP (#C10589, #C10590 and #C10591, respectively, Thermo Fisher Scientific) were used to induce the expression of Rab7‐RFP and GFP or RFP fused to the ER signal sequence of calreticulin and KDEL, which highlight late endosomes and ER, respectively. Viral particles were added at a concentration of 30 per cell for 24–48 h, as recommended by the manufacturer, prior to imaging by live video microscopy or after fixation (see below).

### 2.10 Isolation and characterization of EVs

**FIGURE 1:**
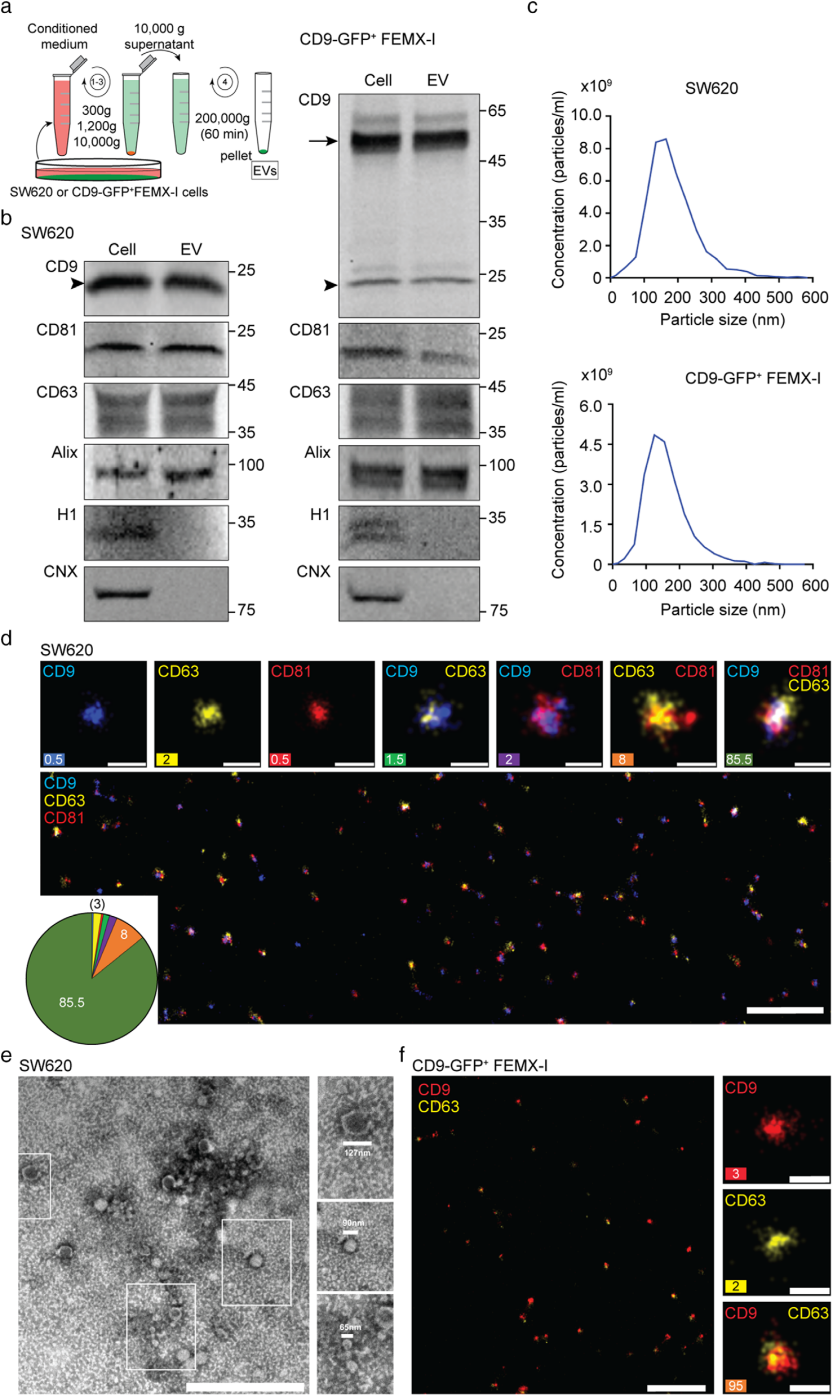
Characterization of EVs derived from SW620 and CD9‐GFP^+^ FEMX‐I cells. (a) Schematic representation of the differential centrifugation protocol used to enrich EVs from the conditioned media of SW620 or CD9‐GFP^+^ FEMX‐I cells. (b) The 200,000 × *g* pellets containing EVs were analysed by immunoblotting for CD9, CD81, CD63, Alix, histone H1 (H1) and calnexin (CNX). Arrow and arrowhead indicate the CD9‐GFP fusion protein and the endogenous CD9, respectively. Molecular mass markers (kDa) are indicated. (c) The size and concentration of EVs were characterized using the ZetaView particle analyser. (d–f) EVs were imaged using dSTORM (d, f) or TEM (e). Upon immunolabelling, the proteins of interest (CD9, CD63 and CD81) were pseudo‐coloured as indicated. Single‐, double‐ and triple‐positive EVs were shown and quantified (d, f) (*n* ≥ 4000 EVs; three independent measurements). The percentage of each is shown and/or presented in the pie chart (d, f). Representative TEM micrograph revealed diverse shape and size of EVs (e, insets). Scale bars, 1 μm (d, f, overview, e), 100 nm (d, f, inset)

EVs were prepared from parental SW620 or CD9‐GFP‐transfected FEMX‐I cells (250,000 cells) cultured in 5 ml serum‐free medium supplemented with 2% B‐27 supplement (#17504044, Thermo Fisher Scientific) on 6‐well plates pre‐coated with 20 μg/ml poly(2‐hydroxyethyl methacrylate) (#P3932, Sigma‐Aldrich) to prevent their attachment as described (Rappa et al., 2017). After 72 h, EVs were enriched by differential centrifugation. Briefly, after low‐speed centrifugations (300 and 1200 × *g*) of conditioned medium, the supernatant fluid was centrifuged at 10,000 × *g* for 30 min at 4°C. The pellet was discarded and the resulting supernatant fluid centrifuged at 200,000 × *g* for 60 min at 4°C (Figure 1a). The pellet was resuspended in 200 μl PBS. The size and concentrations of EVs derived from parental SW620 cells or CD9‐GFP‐transfected FEMX‐I cells were 150 and 120 nm, and 4 × 10^10^ and 2 × 10^10^ particles/ml, respectively, as determined by nanoparticle tracking analysis using ZetaView (software version: 8.05.10; Particle Metrix GmbH, Meerbusch, Germany) according to the manufacturer's protocol. The following parameters were used: 488 nm laser in scatter mode, video duration of 2 s at 30 frames per second for 11 positions along the z‐axis of the analysis window, camera gain of 10, and trace length of 15. EVs were characterized for the presence (CD9, CD81, CD63, and Alix) or absence (histone H1, calnexin) of particular proteins by immunoblotting (see below) according to the guidelines of the International Society for EVs (MISEV2018) (Théry et al., 2018). We have submitted all relevant data of our experiments to the EV‐TRACK knowledgebase (EV‐TRACK, https://evtrack.org/, ID: EV210180) (EV‐TRACK Consortium et al., 2017).

### 2.11 Cell–EV incubation

Parental SW480 cells or those treated to knockdown ORP3, VAP‐A, and VAP‐B as well as FEMX‐I and HeLa cells were seeded at a density of 1 × 10^5^ cells/ml on poly‐D‐lysine‐coated glass‐bottom dishes in a final volume of 1 ml of complete cell medium. Upon attachment, 1 × 10^9^ particles (26 μg proteins) or 2 × 10^9^ particles derived from either SW620 or CD9‐GFP‐transfected FEMX‐I cells in 5 or 10 μl PBS, respectively, were added for 5 h at 37°C. Afterward, cells were processed for immunocytochemistry and subsequent analysis by confocal microscopy. See below for the scratch wound healing assay.

### 2.12 Drug treatments

SW480 cells (1 × 10^5^) were incubated for 5 h with various concentrations of ICZ (2, 5 and 10 μM), H‐ICZ (2, 5 and 10 μM), ketoconazole (10 μM), thapsigargin (1 μM), imipramine (100 μM), OSW‐1 (50 nM), PRR851 (10 μM) or PRR846 (10 μM) as indicated at 37°C. As a control, cells were incubated with DMSO alone. For the thapsigargin rescue experiment, cells were first treated with thapsigargin for 1 h prior to the addition of ICZ (10 μM). They were then incubated for an additional 5 h and processed for immunoisolation and immunofluorescence as described below. In some experiments, EVs were added to recipient cells after a 10‐min pre‐incubation with drugs and further incubated for 5 h in the continuous presence of the drugs. Alternatively, ICZ, H‐ICZ, ketoconazole, PRR851 and PRR846 at the indicated concentrations (1–10 μM) were directly added to detergent cell lysates prepared from SW480 cells (see below) for 30 min on ice with occasional mixing prior to immunoisolation as described below. In the competitive assay, detergent cell lysates were pre‐incubated with 25‐HC at various concentrations (1, 3, 10, 30 or 100 μM) for 30 min on ice, and then ICZ or PRR851 (10 μM) was added for 30 min. For the dephosphorylation experiments, λ‐phosphatase (2000 U/ml) was added to detergent cell lysates and the reaction was supplemented with 2 mM MnCl_2_. The lysates were incubated for 3 h at 30°C. As a control, no enzyme was added. ORP3 was then immunoisolated (see below).

### 2.13 Immunoisolation and immunoblotting

Parental SW480 cells or those deficient in VAP‐A or ORP3 were solubilized in pre‐chilled lysis buffer (0.5% Triton X‐100, 150 mM NaCl, 50 mM Tris‐HCl, pH 8.0, supplemented with Set III protease inhibitor cocktail (#539134, Calbiochem, Merck)) on ice for 30 min, followed by centrifugation (12,000 × *g*) for 10 min at 4°C. In some experiments, 500 mM instead of 150 mM of NaCl or 1% instead of 0.5% Triton X‐100 were used. Immunoisolations were performed on detergent cell lysates using an Ab directed against human ORP3 or VAP‐A followed by Protein G‐conjugated magnetic beads according to the manufacturer's protocols (#130‐071‐101, Miltenyi Biotec, Bergisch Gladback, Germany). Samples were applied to μ Columns (#130‐042‐701, Miltenyi Biotec) for magnetic separation. Materials retained in columns were washed (4x) with 1 ml lysis buffer and rinsed once with 20 mM Tris‐HCl pH 7.5. Pre‐heated (95°C) sodium dodecyl sulphate (SDS) buffer (1% SDS, 50 mM DTT, 1 mM EDTA, 10% glycerol, 0.005% bromophenol blue, 50 mM Tris‐HCl, pH 6.8) was applied to the column to elute the bound fractions. For EV immunoblotting, the SDS buffer was added to the enriched EVs and heated at 95°C for 5 min.

Protein samples were separated onto a 4–20% Tris‐glycine precast gel (#4561095, Bio‐Rad, Hercules, CA, USA) along with the Trident pre‐stained protein molecular weight ladder (#GTX50875, GeneTex, Irvine, CA, USA), and transferred to a nitrocellulose membrane (#88018, Thermo Fisher Scientific) overnight at 4°C. Membranes were incubated in a blocking buffer (PBS containing 1% BSA) for 60 min at room temperature (RT), and then probed with a given primary Ab (Table S1) for 90 min at RT. After three washing steps of 10 min each with PBS containing 0.1% Tween 20 (washing buffer; VWR International), the membranes were incubated with appropriate fluorescein isothiocyanate (FITC)‐coupled secondary Abs diluted in blocking buffer for 30 min at RT. The membranes were rinsed three times with washing buffer, once with distilled and deionized H_2_O and the antigen‐Ab complexes were visualized using the iBright FL1000 imaging system (Thermo Fisher Scientific).

### 2.14 Mass spectrometry

The proteins eluted in Laemmli buffer from ORP3‐ or VAP‐A‐mediated immunoisolation were passed through home‐made suspension trapping filters for clean‐up as previously described (Zougman et al., 2014). The suspension trapping filters were fashioned from 8 × 3 mm punches of QM‐A Whatman quartz discs, stacked above two layers of C18 resin (Empore C18, 3 M) in a 200 μl pipet tip (Zougman et al., 2014). This was filled with 100 mM Tris‐HCl (pH 7.4) in methanol. The samples were solubilized with SDS (4% wt/vol.) containing 20 mM DTT and incubated at 95°C for 5 min, then alkylated with chloroacetamide in the dark for 30 min. Phosphoric acid was added to 3% and the sample transferred to the trapping pipet. Proteins were captured on quartz fibres as a fine dispersion while MS‐incompatible materials passed through the tip. The stack was rinsed and refilled with cold trypsin solution in 50 mM AmBiC and set to digest at 47°C for 2 h. Peptides were eluted with 300 μl of 50% acetonitrile and 0.1% trifluoroacetic acid, lyophilized and stored at ‐20°C. Immunoprecipitated samples were labelled with TMT10plex isobaric labels (Thermo Fisher Scientific), which adds 229.163 Da tags onto lysine residues and the peptide N‐terminus. This afforded precise comparison of recoveries across targeted ORP3 and VAP‐A pull downs. For more details see Supplementary Materials and Methods in Supporting Information section.

For LC/MS/MS, approximately 200 ng of 10‐plex sample mixture was resolved on a 200 cm μPac column (PharmaFulidics, Ghent, Belgium) with a 155‐min gradient propelled by a Proxeon NanoLC 1200 UHPLC. Full data were acquired on a Fusion Orbitrap LUMOS (Thermo Fisher Scientific) running an SPS MS3 protocol as described (Yu et al., 2015). This consists of an orbitrap MS1 scan acquired every 2 s at 60,000 full width at half maximum (FWHM) resolution while full MS2 data is acquired on the Linear Ion trap with a mass isolation window of 0.7 Da. The linear trap is then set to synchronously isolate 10 MS2 fragments retaining the TMT tag to perform high energy activation in the ion routing multipole and injection in the orbitrap where the reporter ions (126‐131 Da) are measured with 50,000 FWHM resolution at 200 Da. A 30‐s dynamic exclusion improved selection of lower abundant precursors. Proteome searches were performed with Proteome Discoverer 2.4 (Thermo Fisher Scientific) and MaxQuant (Cox & Mann, 2008) against the 7/12/2019 human UniprotKB and reverted entry database. With 5 and 10 ppm tolerance for precursor and fragments, respectively, searches assume fixed Cys modifications of 57 Da as well as variable mods of 16 (M) and 80 (S, T) Th. Final discriminant scores were determined by the individual search programs maintaining a false discovery rate (FDR) inferior to 0.02.

### 2.15 Immunocytochemistry

Cells grown on 35‐mm poly‐D‐lysine‐coated glass‐bottom dishes (MatTek Corporation, Ashland, MA, USA) were processed for immunocytochemistry after drug treatment, and/or upon incubation with EVs. Cells were washed with PBS, fixed in 4% paraformaldehyde (PFA) in PBS for 15 min, washed twice with PBS, permeabilized with 0.2% Tween 20 in PBS for 15 min, and blocked with 1% bovine serum albumin (BSA, #97061‐420, VWR International) for 1 h at RT. They were then incubated with primary Abs (Table S1) against specific proteins as indicated in each figure for 60 min at RT, washed twice with PBS, incubated with appropriate fluorescent secondary Abs (Table S2) for 30 min, and washed twice prior to observation. All Abs were diluted in permeabilization buffer containing 1% BSA. To analyse cell morphology, the anti‐CD9 Ab was added to PFA‐fixed, non‐permeabilized cells. Alternatively, cells were stained with ActinGreen^TM^488 ReadyProbes (#R37110, Thermo Fisher Scientific) for 30 min at RT. Nuclei were counterstained with DAPI. In some experiments, after immunolabelling, cells were incubated with filipin at 50 μg/ml working concentrations for 2 h at 20°C and then washed twice with PBS. Cells were imaged in PBS using either the Nanoimager high‐resolution microscope (ONI, Oxford, UK) with 100X oil‐immersion objective or the Nikon A1R+ inverted confocal microscope (Nikon, Melville, NY, USA) with a 60X Apo‐TIRF oil‐immersion objective. All images were acquired under the same microscope settings for subsequent calculations of mean fluorescence and recorded using Nanoimaging (ONI) or NIS Elements software (Nikon). Fluorescence signal was quantified using Fiji software (Schindelin et al., 2012). To measure the cytoplasmic‐associated GFP signal upon incubation of cells with CD9‐GFP^+^ EVs, regions of interest (ROIs) were drawn around the cytoplasm of SUN2‐immunolabelled cells, excluding the nuclear compartment, and the ‘measure’ function on Fiji was applied to determine the fluorescent signal. The total cell fluorescence was then calculated as total cell fluorescence = integrated density – (area × background mean fluorescence). To determine the nuclear EV‐derived fluorescent materials, ROIs were plotted along the nucleus and an automatic threshold generated by Fiji was applied as described (Rappa et al., 2017). Positive signals were counted using the ‘analyse particle’ function and presented as dot plots.

### 2.16 Stochastic optical reconstruction microscopy

Three‐dimensional (3D) direct stochastic optical reconstruction microscopy (dSTORM) was applied either on cells or EVs. In the case of cells, SW480 cells were seeded at a density of 5 × 10^4^ cells/ml on 8‐well glass‐bottom (#1.5H thickness) μ‐slides (#80827, Ibidi, Fitchburg, WA, USA). They were treated without or with 10 μM drugs (ICZ, PRR851) for 5 h, PFA‐fixed and permeabilized with 0.2% Tween 20 in PBS for 15 min each. They were blocked with 1% BSA, 0.2% Tween 20 in PBS for 1 h and then double labelled with primary Abs against ORP3 or Rab7 for 1 h at RT. Appropriate secondary Abs conjugated to Atto 488 (#610‐152‐121, Rockland Immunochemicals, Limerick, PA, USA) or Alexa Fluor647 (#A‐21246, Thermo Fisher Scientific) were applied for 30 min. Cells were post‐fixed in 4% PFA. Cells were imaged using a Nanoimager high‐resolution microscope with 100X oil‐immersion objective. A STORM‐imaging buffer was used to attain the ‘blinking’ event of the dyes required in imaging process. It is a random activation of the dyes from an off or dark state to an on or emission state and back, thus allowing single particle illumination (Rust et al., 2006). The buffer consists of glucose oxidase (0.5 mg/ml, #G2133, Sigma‐Aldrich), catalase (40 μg/ml, #C1345, Sigma‐Aldrich), glucose (10% m/v), and 2‐mercaptoethylamine (10 mM, #30070, Sigma‐Aldrich) diluted in Tris‐buffered saline (50 mM Tris‐HCl, pH 8.0, 10 mM NaCl). Prior to imaging, channel and 3D mapping calibrations were performed using 0.1 μm TetraSpeck beads (#T7279, Thermo Fisher Scientific). Lasers (488 and 640 nm) were set to 100% power and 10,000 frames were acquired per channel sequentially, collecting about 100–250 particles per frame. The thickness of acquired sections is 1 μm. Drift correction was applied to each image using the built‐in function of ONI's NimOS software. ROIs were drawn on the entire areas of cytoplasm, perinuclear zone and NEI then rendered in 3D image using Imaris software by Bitplane (Concord, MA, USA). A modelling of the protein of interests was generated using the ‘spots’ and ‘surface’ functions. Then, the distance between two structures (i.e., ORP3 or Rab7 positive) were calculated using the ‘statistics’ function. ROIs were made also around the plasma and nuclear membranes to evaluate the volume of Rab7^+^ structures in the pericellular and perinuclear zones, respectively. A filter was applied to show structures with a volume greater than 20 nm^3^.

EVs prepared from SW620 or CD9‐GFP^+^ FEMX‐I cells (3.5 × 10^7^ and 1.1 × 10^8^ particles, respectively) were immunolabelled overnight at 4°C using a cocktail of fluorescently labelled anti‐tetraspanin Ab against CD9 (Atto488 mouse anti‐human monoclonal; FL‐REA‐EV‐CD9‐Atto488), CD63 (Cy3B^TM^ mouse anti‐human monoclonal; FL‐REA‐EV‐CD63‐Cy3b), and CD81 (Alexa Fluor^®^647 rat anti‐human monoclonal; FL‐REA‐EVCD81‐AF647). CD9‐Alexa Fluor^®^647 was used for CD9‐GFP^+^ EVs. In the latter case, we did not use the green channel to avoid crosstalk between the immunostaining and the GFP signal. Stained EVs were loaded and captured on the surface of a PEG‐Biotin functionalized microfluidic chip included in the EasyVisi Single‐Extracellular Vesicle Characterization kit from ONI (beta version 1.0, Oxford Nanoimaging, UK). Surface preparation, removal of unbound Ab, and crosslinking of EVs to the chip surface, including all wash steps, were performed using the EasyVisi kit and automated using a Roboflow microfluidic sample preparation platform (ONI). Freshly prepared BCubed STORM‐imaging buffer (ONI) was added to each lane on the microfluidics chip before imaging. Single‐molecule fluorescence data consisting of 2000 frames per channel, was sequentially acquired using the Nanoimager S Mark II with laser power set to 45, 50, and 50% for the 640, 560, and 488 lasers, respectively. An Olympus 1.4NA 100x oil immersion super apochromatic objective was used with angle of illumination set to 52.5°. Channel mapping was calibrated at the start of the imaging session using 0.1 μm Tetraspeck beads (Thermo Fisher Scientific). Data was processed on NimOS software (version 1.18; ONI). To identify EV subpopulations that express one, two, or three markers, single‐molecule data was analysed using algorithms developed by ONI via their online localization microscopy data analysis platform beta‐released named CODI (https://alto.codi.bio/, releases 0.16.0 to 0.14.1; 9 March 2021 to 28 April 2021). The analysis workflow of EV data included filtering, drift correction, and subsequent clustering using hierarchical density‐based clustering algorithms for single‐EV analysis.

### 2.17 Incubation of fluorescent drug

ER‐RFP‐expressing SW480 cells were incubated with the green‐fluorescent compound PRR898 (2 μM) for 1 h, then washed with RPMI medium and further incubated for the indicated time. They were then fixed with 4% PFA and imaged using the Nanoimager microscope.

### 2.18 Time‐lapse video microscopy

SW480 cells expressing ER‐GFP and Rab7‐RFP or FEMX‐I cells expressing VAP‐A‐GFP and Rab7‐RFP grown on 35‐mm poly‐D‐lysine‐coated glass‐bottom dishes (Mattek) were treated with ICZ or H‐ICZ (see above) and then imaged live under 5% CO_2_ atmosphere at 37°C. For SW480 cells, time‐lapse videos were recorded every 20 s for a period of 5 min using the Nanoimager microscope, whereas the images of FEMX‐I cells were acquired every 60 s for a 10 min‐period using the A1R+ confocal microscope. Videos were made at a playing speed of one or three frames per second as indicated, and a single *x*‐*y* optical section (0.2 μm) was acquired at a given time. The tracking and the velocity of Rab7^+^ structures were evaluated using TrackMate plugin available in the Fiji software (Tinevez et al., 2017).

### 2.19 Electron microscopy

The EVs recovered from 200,000 × *g* pellet were prepared for transmission electron microscopy (TEM) using negative staining. A 5‐μl aliquot of EV suspension was applied onto 200‐mesh carbon‐coated EM grids. After subsequent washing steps with PBS, the grids were fixed for 5 min in 1% glutaraldehyde in PBS. The sample was negatively contrasted with 2% aqueous solution of phosphotungstic acid. The grids were viewed in a JEOL JEM 1400 Plus electron microscope operated at 80 kV. The images shown were prepared using Adobe Photoshop software.

### 2.20 Cell migration assay

Cell migration was evaluated by a scratch wound healing assay. SW480 cells were seeded at concentration of 2 × 10^5^ cells/well in 12‐well plates. Once the cells reached full confluence, the wound healing assay was performed by scratching the cell monolayer with a sterile pipette tip. The cells were washed to remove detached cells, and then treated without or with 10 μM drugs (ICZ or PRR851) for 5 h in the absence or presence of SW620 cell‐derived EVs (7.5 × 10^8^ particles/ml or 20 μg protein/ml). Images of scratch wounds were captured using an inverted microscope (Olympus IX70, Segrate, Italy) before (0 h) and after (5 h) treatment. The wound areas were measured with ImageJ (Schindelin et al., 2012). At least 10 fields/conditions were analysed from two independent experiments. For each condition, the Wound Area at 0 h was considered as the 100%.

### 2.21 Growth inhibition assay

Cells were seeded at a density of 4 × 10^3^ cells on 96‐well plates and incubated overnight to allow their adhesion. They were then treated with ICZ, H‐ICZ, ketoconazole, PRR851 and PRR846 at different concentrations (1, 3, and 10 μM) for a given time (24, 48 and 72 h), as indicated. DMSO was used as control. At the appropriate time, a CellTiter96 AQueous One Solution Cell Proliferation Assay (1:5 dilution; #G3580, Promega) was added for 2 h at 37°C. The reagent utilizes the biochemical reaction of a tetrazolium compound [3‐(4,5‐dimethylthiazol‐2‐yl)‐5‐(3‐carboxymethoxyphenyl)‐2‐(4‐sulfophenyl)‐2H‐tetrazolium, MTS] to produce a coloured, soluble formazan product, that is, proportional to the number of live cells. The absorbance value was measured at 490 nm using the Varioskan Flash plate reader (Thermo Fisher Scientific).

### 2.22 Statistical analysis

All experiments were carried out at least in triplicate, with the exception of the cell migration assay, which was performed in duplicate. The error bars in the graphical data represent the mean ± standard deviation (S.D.) or standard error of the mean (S.E.M.) as indicated in the figure legends. Statistical significance was determined using a two‐tailed Student's t‐test, and *P* values < 0.05 were considered significant. All graphs were created using GraphPad Prism 8.

## 3 RESULTS

### 3.1 Itraconazole inhibits the EV‐induced pro‐metastatic morphological transformation

Numerous studies have demonstrated the pro‐malignant impact of EVs derived from cancer cells on the surrounding environment, including cell transformation (Zomer et al., 2015; Zomer & van Rheenen, 2016). Non‐metastatic elongated SW480 colon cancer cells are an excellent model for determining EV‐induced transformation. Compared to SW480 cells, highly metastatic SW620 cells have a more rounded shape, associated with amoeboid motility and high plasma membrane blebbing activity (de Toledo et al., 2012; Schillaci et al., 2017). These pro‐metastatic properties can be transferred to SW480 cells by EVs derived from SW620 cells (Schillaci et al., 2017). To assess whether these cellular alterations are related to the nuclear transfer of the EV cargo, we exposed SW480 cells as well as those silenced for VAP‐A, VAP‐A homolog, VAP‐B, and ORP3 for 5 h to EVs prepared from SW620 cells. The lack of VOR complex proteins should impair the nuclear transfer of EV cargo, and potentially the malignant transformation (Rappa et al., 2017; Santos et al., 2018).

The silencing of VAP‐A, VAP‐B and ORP3 in SW480 cells was achieved using the short hairpin RNA (shRNA) technology (Santos et al., 2018). As a control, SW480 cells were stably transfected with a scrambled shRNA plasmid. Their reduction was confirmed and quantified by immunofluorescence followed by confocal laser scanning microscopy (CLSM) analysis and immunoblotting (Figure S1a‐c). EVs released by SW620 cells were enriched by differential centrifugation of conditioned media and characterized by immunoblotting, nanoparticle tracking, dSTORM and TEM analyses. They were positive for membrane proteins CD9, CD81 and CD63 as well as for cytoplasmic protein Alix, which are characteristic constituents of EVs, while nuclear histone H1 and ER‐resident protein calnexin were absent (Figure 1b), in line with other studies (Jeppesen et al., 2019; Théry et al., 2001; Théry et al., 2018). EVs ranged in size from 50 to 350 nm, suggesting the presence of exosomes and microvesicles (Figure 1c). Consistent with these observations, we visualized by dSTORM, a single molecule super‐resolution technique, various EV subpopulations as immunolabelled with tetraspanin proteins (Figure 1d). The triple positive (CD9, CD63, and CD81) EVs represent the major subpopulation in agreement with others (Kowal et al., 2016). The CD9^–^CD63^+^CD81^+^ EVs constitute the second abundant subpopulation, while EVs labelled only with one of them represent a minor fraction. The heterogeneity in EV size is also confirmed by TEM where the diameter of EVs varies from small (< 60 nm), intermediate (60–90 nm), and large (> 90 nm) (Figure 1e), in agreement with other studies (Chen et al., 2019; Endzeliņš et al., 2018; Popēna et al., 2018).

**FIGURE 2:**
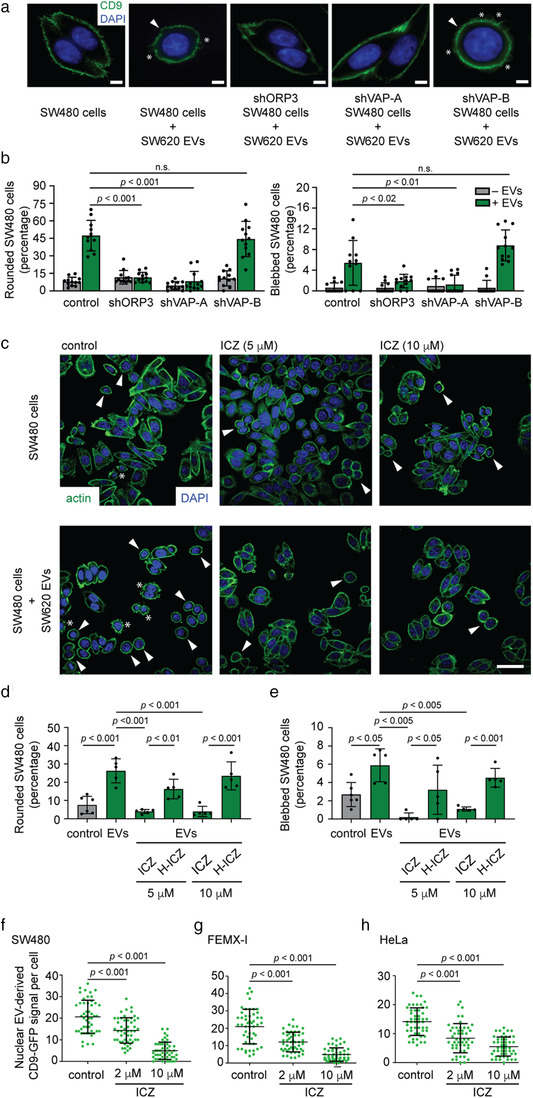
Silencing VOR complex or the ICZ treatment inhibits EV‐induced pro‐metastatic morphological transformation and nuclear transfer of EV cargo in SW480 cells. (a, b) Non‐ metastatic SW480 cancer cells (scrambled shRNA control) and those treated to knock down ORP3 (shORP3), VAP‐A (shVAP‐A) and VAP‐B (shVAP‐B) were incubated for 5 h without or with EVs (1 × 10^9^ particles/ml) derived from metastatic SW620 cells prior to CD9 immunolabelling. Nuclei were visualized with DAPI and cell morphology was analysed by CLSM (a). Bar graphs show the percentage of cells harbouring a rounded morphology (left) or membrane blebbing (right) upon exposure to EVs (b). (c‐e) SW480 cells were pre‐treated with DMSO (control) or 5 and 10 μM ICZ (c‐e) or H‐ICZ (d, e) for 10 min prior to the addition of SW620 cell‐derived EVs and then incubated for 5 h. Cells were stained for actin (ActinGreen, green) and nuclei (DAPI, blue) (c). Bar graphs show the percentage of cells harbouring a rounded morphology (d) or membrane blebbing (e). At least 100 cells were evaluated per condition and independent experiment (b, *n* = 11–12; d, e, *n* = 5–6). Arrowheads and asterisks indicate the rounded cell morphology and the membrane blebbing induced by EVs. (f‐h) SW480 (f), FEMX‐I (g) and HeLa (h) cells were pre‐treated with DMSO (control) or 2 and 10 μM ICZ for 10 min prior to 5‐h incubation with FEMX‐I cell‐derived CD9‐GFP^+^ EVs (1 × 10^9^ particles/ml) in the absence or presence of drugs. Cells were fixed and immunolabelled for SUN2. The CD9‐GFP signal in the nuclear compartment was analysed by CLSM and quantified. Independent values for each cell (*n* = 50 per condition) from a representative experiment are displayed (f‐h). In all cases, means ± S.D. are shown. *P* values are indicated. The quantification of three independent experiments for each cell line is presented in Table S4. N.s., not significant. Scale bars, 5 (a) or 20 (c) μm

Upon exposure to SW620 cell‐derived EVs, SW480 cells were immunolabelled for CD9 (or labelled with ActinGreen^TM^488 ReadyProbes) and observed by CLSM. Their shape and membrane blebbing changed significantly as scored by two independent investigators (Figure 2a, b), in agreement with a previous report from one of our labs (Schillaci et al., 2017). This phenotype was abolished upon ORP3 or VAP‐A knockdown. These events are specific for VOR complex proteins since VAP‐B silencing did not block them (Figure 2a, b). These data suggest that the nuclear transfer of the EV cargo is involved in the aforementioned phenotype.

We assessed whether ICZ could interfere with EV‐mediated pro‐metastatic morphological transformation. We pre‐treated SW480 cells with 5 or 10 μM ICZ or its major metabolite H‐ICZ for 10 min prior to incubation with SW620 cell‐derived EVs for 5 h. The drugs were kept during EV incubation. As a negative control, cells were incubated with the solvent vehicle only (i.e., 0.1% DMSO). Similar to the ORP3 or VAP‐A knockdown, ICZ prevented the rounding and/or blebbing of cells (Figure 2c‐e). H‐ICZ did not avert these EV‐mediated changes. Indeed, H‐ICZ partially reduced the number of rounded and blebbed SW480 cells under native conditions, that is, without the addition of EVs (Figure S2). These data suggest that ICZ inhibited the nuclear transfer of the EV cargo, thus interfering with the malignant transformation.

To visualize the nuclear transfer of the EV cargo, and the impact of ICZ on it, we used an assay previously developed in our laboratory, where bioengineered fluorescent EVs are incubated with host cells prior to quantification of internalized cytoplasmic and nuclear fluorescent signals (Rappa et al., 2017; Santos et al., 2018). The EVs were enriched from conditioned media of CD9‐GFP^+^ FEMX‐I cells and characterized such as those described above (Figure 1b, c, d, f). The human melanoma cell line FEMX‐I has been used to identify the VOR complex (Santos et al., 2018), and the morphology of EVs derived from it has already been documented by CD63‐immunoelectron microscopy (Rappa et al., 2013b). Three cell lines, SW480, FEMX‐I and HeLa cells, were pre‐treated without or with 2 or 10 μM ICZ for 10 min prior to incubation with CD9‐GFP^+^ EVs for 5 h. Subsequently, fixed‐cells were immunolabelled for SUN domain‐containing protein 2 (SUN2) to highlight the nuclear compartment. They were analysed by CLSM through serial *x*‐*y* optical sections (25–30 sections per cell, 0.2 μm each) covering the entire cell of interest. Fluorescent cytoplasmic and nuclear signals (i.e., GFP) were quantified. For all cell lines, no reduction in the overall intensity of cytoplasmic GFP in the host cells were found suggesting that ICZ does not block EV endocytosis (Figure S3a‐c), which is consistent with a recent report (Lin et al., 2018). In contrast, discrete nuclear GFP punctate signals distributed throughout the nucleoplasm decreased significantly upon incubation with 2 or 10 μM ICZ (Figure 2f‐h; Table S4; see also Figure S3d), indicating that ICZ interferes with the nuclear transfer of EV cargo. Overall, this first set of experiments demonstrated that ICZ phenocopies the silencing of ORP3 and VAP‐A by inhibiting EV‐mediated cell transformation. It remains to be determined whether the observed effects are related to the impact of ICZ on the integrity of the VOR complex, and possibly the absence of late endosomes in the nucleoplasmic reticulum.

### 3.2 Identification of ICZ as a VOR complex inhibitor

To evaluate whether ICZ interfered with the interaction of ORP3 with VAP‐A and/or Rab7, we set up a co‐immunoisolation protocol based on paramagnetic bead separation (Santos et al., 2018). Two distinct Abs against human ORP3, the antiserum (AS) A304‐557A and monoclonal Ab D‐12, were used (Figure S4a). By immunoblotting SW480 cell lysate prepared with a lysis buffer containing 0.5% Triton X‐100 and 150 mM NaCl, both Abs recognized a broad ORP3 immunoreactive band (∼95 to 100 kDa; Figure S4b). The ORP3 immunoreactivity often appeared as a very close doublet, which could correspond to the hyper‐ (∼101 kDa) and hypo‐phosphorylated (∼97 kDa) forms of the protein (Weber‐Boyvat et al., 2015). Coupled with magnetic beads conjugated to G protein, Ab D‐12 could selectively pull down ORP3 together with VAP‐A and Rab7 as observed by immunoblotting (Figure S4c) and liquid chromatography/tandem mass spectrometry (LC/MS/MS) analysis of D‐12 immunoisolated materials, where peptides of three proteins were recovered (Figure S4d; Table S5).

**FIGURE 3:**
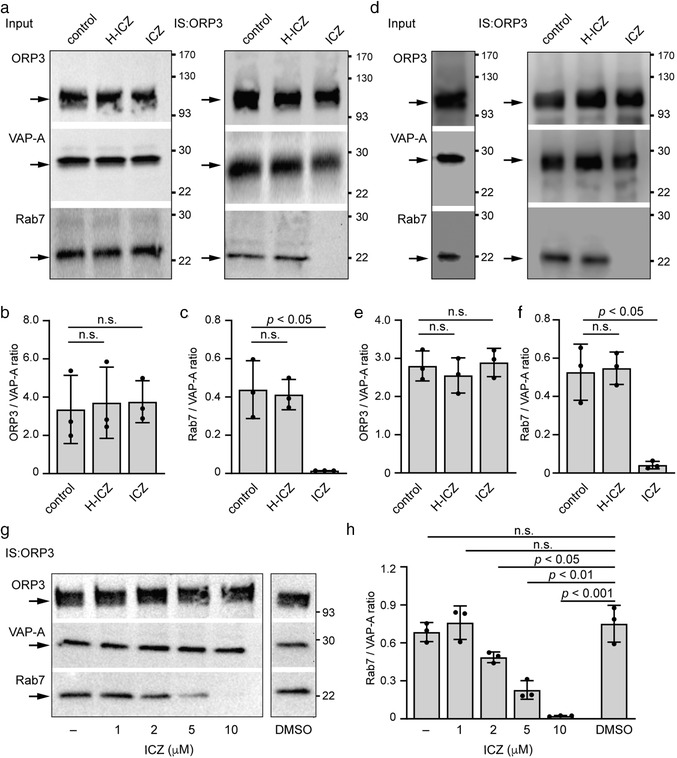
ICZ disrupts the binding of Rab7 to ORP3 and VAP‐A. (a‐c) SW480 cells were incubated with either DMSO (control), 10 μM ICZ or H‐ICZ for 5 h, solubilized and subjected to immunoisolation (IS) using anti‐ORP3 Ab followed by Protein G‐coupled magnetic beads. The input (1/50) and entire bound fractions were probed by immunoblotting for ORP3, VAP‐A and Rab7 (a). The ratio of protein immunoreactivities of the indicated pairs was quantified (b, c, *n* = 3). (d–f) The SW480 cell detergent lysate was divided in three aliquots and incubated with DMSO (control), 10 μM ICZ or H‐ICZ for 30 min on ice, and then subjected to IS with anti‐ORP3 Ab and immunoblotting (d). The input (1/50) and bound fractions were analysed. The ratio of protein immunoreactivities of the indicated pairs was quantified (e, f, *n* = 3). (g, h) SW480 cells were exposed to different ICZ concentrations as indicated for 5 h, solubilized and subjected to IS and immunoblotting (g). As controls, no solvent (–) or DMSO (0.1%) were used. The ratio of protein immunoreactivities of the indicated pairs was quantified (h, *n* = 3). For all immunoblots, molecular mass markers (kDa) are indicated, and arrows point to the proteins of interest. The mean ± S.D. are shown. *P* values are indicated. N.s., not significant

Upon 5‐h incubation of SW480 cells with ICZ or H‐ICZ (10 μM), cells were solubilized prior to immunoisolation using anti‐ORP3 D‐12 Ab. As a negative control, cells were incubated with the solvent vehicle only. The immunoisolated materials were probed by immunoblotting for ORP3, VAP‐A and Rab7. ICZ, but not H‐ICZ, completely abolished the interaction of ORP3/VAP‐A with Rab7, while the ratio of ORP3 and VAP‐A was unaffected (Figure 3a‐c), suggesting that the potential binding of ICZ to ORP3 interferes with ORP3–Rab7 interaction. Since the observed effect of ICZ could be caused by various intracellular factors, we added the drugs to detergent cell lysates in the cold, instead of living cells, prior to ORP3 immunoisolation. The interaction of ORP3/VAP‐A with Rab7 in the lysates was also disrupted by ICZ (Figure 3d‐f), suggesting that this compound acts directly on the VOR complex. The inhibition of ORP3/VAP‐A–Rab7 interaction was also observed at lower concentrations (i.e., 2 and 5 μM ICZ), although less effectively (Figure 3g, h).

**FIGURE 4:**
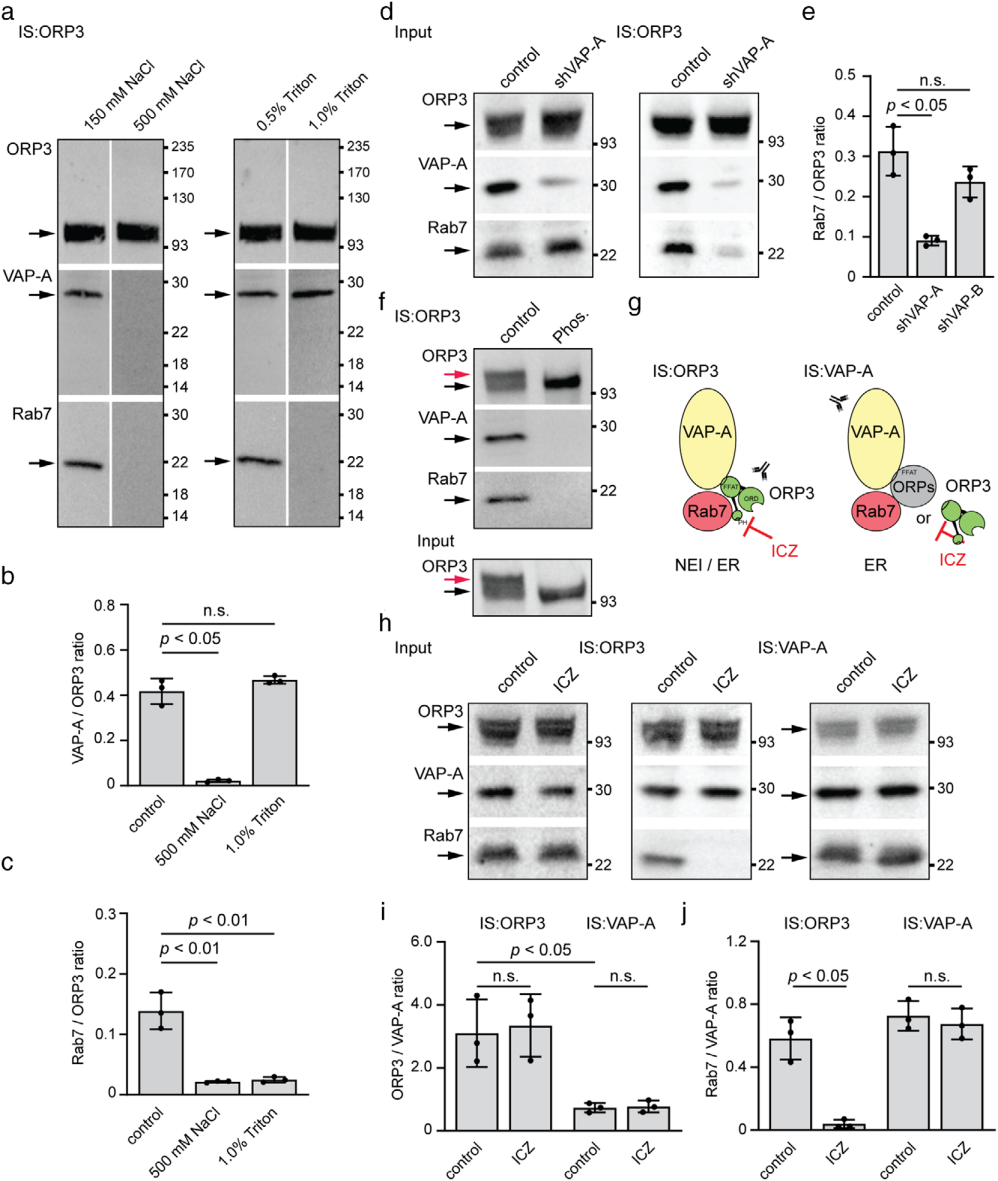
ICZ disrupts the binding of ORP3 to Rab7, but not to VAP‐A. (a‐c) SW480 cells were solubilized in the presence of different concentrations of NaCl or Triton X‐100 as indicated prior to being subjected to immunoisolation (IS) using anti‐ORP3 Ab and Protein G‐coupled magnetic beads. The bound fraction was probed by immunoblotting for ORP3, VAP‐A and Rab7 (a). The ratio of protein immunoreactivities of the indicated pairs was quantified (b, c, *n* = 3). (d, e) Detergent lysates prepared from parental (scrambled control) and VAP‐A‐silenced (shVAP‐A) SW480 cells were processed as described in panel a. The input (1/50) and bound fractions were analysed by immunoblots (d). The ratio of protein immunoreactivities of the indicated pairs was quantified (e, *n* = 3; see Figure S4e for corresponding blot of SW480 shVAP‐B data). (f) The detergent lysate prepared from SW480 cells was divided into two aliquots and incubated without (control) or with λ‐phosphatase (Phos.) for 3 h prior to ORP3 immunoisolation and immunoblotting. It should be noted that there were no slower‐migrating ORP3 immunoreactive species after phosphatase treatment. (g) Diagram illustrating the tripartite protein complex interactions between VAP‐A (yellow), Rab7 (red) and ORP3 (green) or another oxysterol binding protein (OSBP)‐related protein (ORPs, grey) at the level of nuclear envelope invagination (NEI) and/or endoplasmic reticulum (ER). The PH, ORD and FFAT motifs found in ORP3 are indicated. Abs used for the IS are directed against either VAP‐A or ORP3. Note the possible molecular interaction target of ICZ. (h‐j) SW480 cells were incubated with DMSO alone (control) or 10 μM ICZ for 5 h, solubilized and subjected to IS using either anti‐ORP3 or anti‐VAP‐A Ab followed by Protein G‐coupled magnetic beads. The input (1/50) and entire bound fractions were probed by immunoblotting for ORP3, VAP‐A and Rab7 (h). The ratio of protein immunoreactivities of the indicated pairs was quantified (i, j, *n* = 3). For all immunoblots, molecular mass markers (kDa) are indicated. The arrows point to the proteins of interest while the red arrow (f) indicates specifically the hyperphosphorylated form of ORP3. For all graphs, the mean ± S.D. are shown. *P* values are indicated. N.s., not significant

The immunoisolation data obtained from ICZ‐treated cell lysates suggest a direct interaction between the three VOR complex proteins. In agreement with this conclusion, when cells were solubilized in the presence of a high‐ionic strength buffer containing 500 instead of 150 mM NaCl, which disrupts most ionic protein interactions, VAP‐A and Rab7 were not co‐immunoisolated with ORP3 (Figure 4a‐c). Increasing the concentration of the Triton X‐100 detergent from 0.5 to 1.0% in the solubilization buffer prevented the co‐immunoisolation of Rab7 with ORP3, but did not disrupt the binding of VAP‐A to ORP3, reflecting the stronger binding of ORP3 with the integral membrane protein VAP‐A than with the lipid‐anchored Rab7. These data suggest that ICZ perturbed the interaction of ORP3/VAP‐A with Rab7, which eventually impedes the entry of late endosomes into NEI (see below).

To further dissect the ORP3/VAP‐A–Rab7 interaction, we used VAP‐A‐deficient SW480 cells. These cells, as well as control cells transfected with a scrambled shRNA plasmid, were solubilized and then subjected to ORP3 immunoisolation. Immunoblotting of ORP3, VAP‐A and Rab7 showed that VAP‐A is required for the interaction of ORP3 with Rab7 (Figure 4d, e). In contrast, when a similar experiment was carried out with cells deficient in VAP‐B (Figure S1a‐c; Figure S4e), the ORP3–Rab7 interaction remained intact (Figure 4e). This information suggests that VAP‐A could be involved either in a conformational change of ORP3 that allows its subsequent interaction with Rab7, or that both VAP‐A and ORP3 are required for Rab7 binding. It is documented that the interaction of ORP3 with VAP‐A is strongly stimulated by the hyperphosphorylation of ORP3 (Weber‐Boyvat et al., 2015). In agreement with the latter, λ‐phosphatase treatment of detergent SW480 cell lysates prior to ORP3 immunoisolation completely suppressed the interaction with VAP‐A as well as with Rab7 (Figure 4f), suggesting that phosphorylated ORP3–VAP‐A interaction is a prerequisite for Rab7 binding. The efficacy of phosphatase treatment was validated by a perceptive change in the mobility of ORP3.

The role of ORP3 in the VOR complex was then investigated using ORP3‐deficient cells (Figure S1a‐c; Figure S4f, g). The immunoisolation was performed with anti‐VAP‐A Ab (Figure 4g). As described for ORP3‐driven immunoisolation, we observed under native conditions a co‐isolation of the three VOR complex proteins by immunoblotting (Figure 4h; Figure S4f) or LC/MS/MS analysis (Figure S4d; Table S5). The large difference in ORP3/VAP‐A ratios observed upon VAP‐A‐ versus ORP3‐ driven immunoisolation (Figure 4h, i) is indicative of other types of VAP‐A interactions beside the VOR complex (Figure 4g). In line with the indirect interaction of VAP‐A‐Rab7 via other OSBP‐related proteins, ORP3 knockdown did not impede the co‐isolation of VAP‐A and Rab7 (Figure S4f, g). Of note, cell exposure to ICZ did not affect the binding of Rab7, as observed by VAP‐A‐driven immunoisolation opposed to the ORP3‐driven immunoisolation (Figure 4h‐j; Figure S4f, g), suggesting that ICZ blocks the interaction of ORP3 and Rab7, but not that of other OSBP proteins and Rab7 (Figure 4g).

In summary, this second set of experiments suggests that there are at least two distinct sets of VAP‐A‐Rab7 interactions – the established one, ICZ‐insensitive, possibly observed at the ER membrane where it could participate in the maturation and positioning of late endosomes (Eden, 2016) and involve the cholesterol sensor ORP1L (Rocha et al., 2009), and the ICZ‐sensitive one involving ORP3, which regulates the entry of late endosomes into nucleoplasmic reticulum (Figure 4g) (Santos et al., 2018). It remains to be established whether the latter occurs at the gate of, and/or within, type II NEI.

### 3.3 Itraconazole impedes the localization of Rab7^+^ late endosomes in the nucleoplasmic reticulum

**FIGURE 5:**
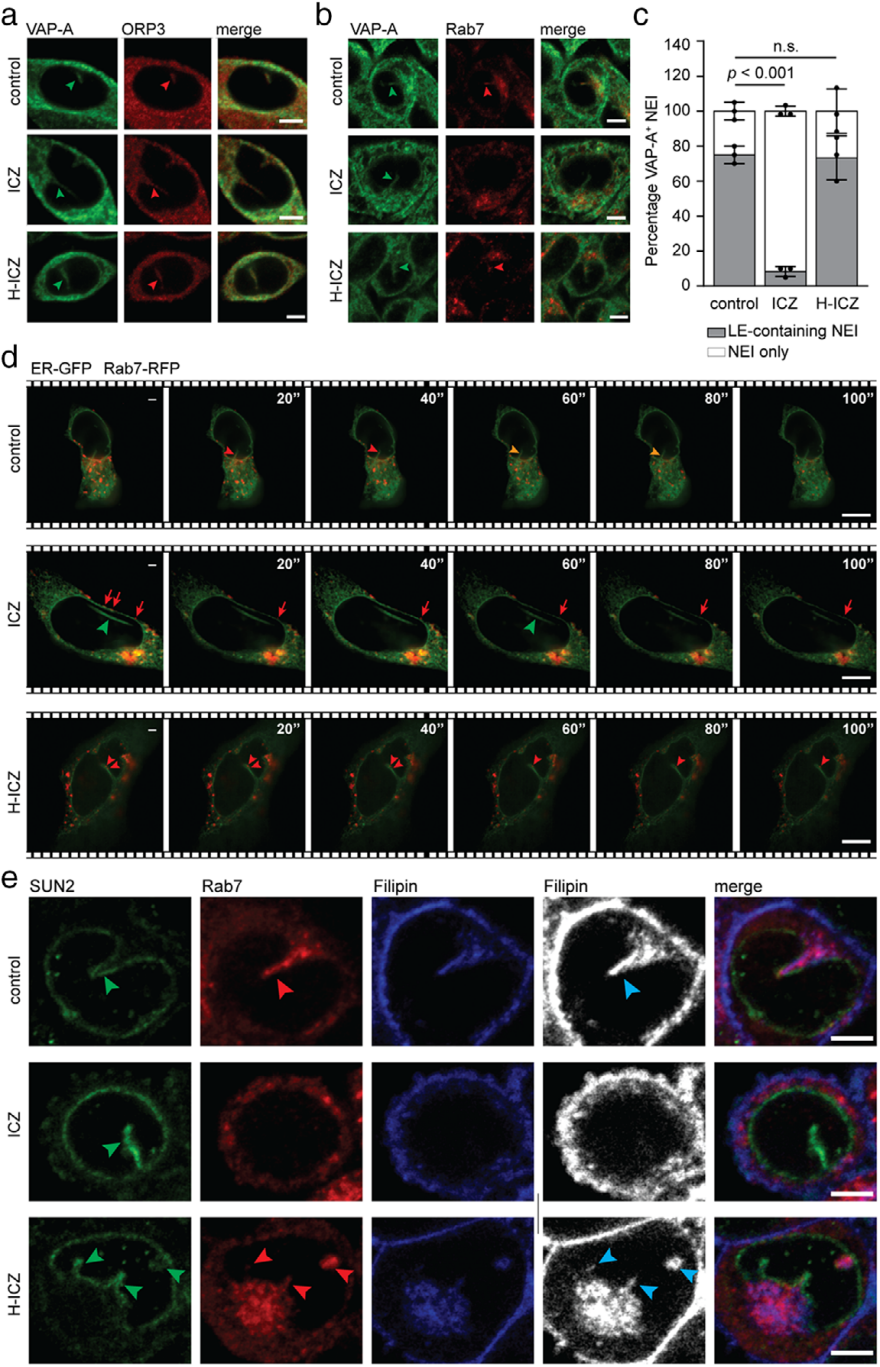
ICZ inhibits the localization of Rab7, but not ORP3, in type II NEI. (a‐c) SW480 cells were incubated with DMSO (control), 10 μM ICZ or H‐ICZ for 5 h prior to double immunolabelling for VAP‐A and ORP3 (a) or VAP‐A and Rab7 (b). Cells were observed by CLSM. Arrowheads indicate the presence of ORP3 or Rab7 (red) in VAP‐A^+^ NEI (green) (a, b). Note the absence of Rab7 in VAP‐A^+^ NEI of ICZ‐treated cells. Bar graph shows the percentage of VAP‐A^+^ NEI containing Rab7^+^ late endosomes (LE) (c). The means ± S.D. are shown. At least 20 VAP‐A^+^ NEI were evaluated per experiment (*n* = 3). *P* values are indicated. N.s., not significant. (d) SW480 cells co‐expressing ER‐GFP and Rab7‐RFP were treated with DMSO (control) or a drug (10 μM, 5 h) as indicated, and then recorded by time‐lapse video microscopy every 20 s for a period of 5 min. Elapsed time is indicated in the top right corner. Red and yellow arrowheads show Rab7‐RFP^+^ LE moving along ER‐GFP^+^ NEI in the absence (control) or presence of H‐ICZ. Green arrowhead points to the ER‐GFP^+^ NEI, that is, devoid of Rab7‐RFP in the presence of ICZ, while red arrows indicate the Rab7‐RFP moving along the nuclear membrane under the same conditions. (e) SW480 cells were treated as above prior to double immunolabelling for SUN2 and Rab7, and filipin staining. Black and white images are presented to better show filipin‐labelled cholesterol (cyan arrowhead). Green and red arrowheads point to SUN2 and Rab7 in NEI. For all, single cell images are shown. Scale bars, 5 μm

To determine the impact of ICZ on the subcellular localization of Rab7^+^ late endosomes, particularly with respect to their entry into the nucleoplasmic reticulum, SW480 cells were treated with either 10 μM ICZ or H‐ICZ, or DMSO as a negative control, for 5 h and double immunolabelled for VAP‐A and ORP3 or Rab7. Cells were observed by CLSM. Although the occurrence of ORP3 in VAP‐A^+^ NEI was unaffected by drugs (Figure 5a), Rab7 was absent in the presence of ICZ, but not of H‐ICZ (Figure 5b). The reduction in total Rab7^+^ VAP‐A^+^ NEI was approximately 65% (Figure 5c). To obtain information on the transport of Rab7^+^ late endosome in the nucleoplasmic reticulum, we co‐expressed in SW480 cells the Rab7‐RFP fusion protein to label late endosomes and chimeric GFP fused to the ER signal sequence of calreticulin and KDEL to label peripheral ER and nuclear envelope. Cells were treated with drugs and monitored by live video microscopy. In control cells incubated with DMSO and those treated with H‐ICZ, fluorescent Rab7 could be detected in ER‐GFP^+^ NEI in addition to the cytoplasm (Figure 5d). Videos revealed the movement of Rab7‐RFP^+^ late endosomes in both subcellular compartments (Videos S1 and S2). In contrast, no Rab7‐RFP was detected in the nucleoplasmic reticulum of ICZ‐treated cells (Figure 5d, green arrowhead; Videos S3 and S4). Under these conditions, the Rab7‐RFP motility along the nuclear rim and in the cytoplasm was not abrogated, although its movement appeared somehow slower (Figure 5d, arrows; Figure S5a, b; Videos S3 and S5). Quantification of Rab7^+^ late endosome velocity in the cytoplasmic compartment was reduced in ICZ‐treated cells compared with the native state and in the presence of H‐ICZ (Figure S5c), which is consistent with previous studies (Lebrand et al., 2002; Loubery et al., 2008). Similar data were obtained with Rab7‐RFP^+^VAP‐A‐GFP^+^ FEMX‐I cells showing that the impact of ICZ on the entry and/or retention of late endosomes into the NEI is not limited to a single cell type (Figure S5d; Videos S6‐S8).

To ascertain that Rab7 labelled late endosomes rather than being uncoupled from the endosomal system, we evaluated whether any organelles, notably those enriched in membrane cholesterol, were found in nucleoplasmic reticulum by employing filipin, a naturally fluorescent polyene antibiotic that selectively binds to unesterified cholesterol in biological membranes (Kinsky, 1970). Drug‐treated cells were immunolabelled for both SUN2 and Rab7 prior to filipin staining. A strong filipin signal, often overlapping with Rab7, was detected in SUN2^+^ NEI in control cells and those treated with H‐ICZ (Figure 5e). In contrast, both filipin and Rab7 signals were excluded from NEI in ICZ‐treated cells, suggesting that cholesterol‐containing organelles such as Rab7^+^ late endosomes were absent therein (Figure 5e). In agreement with earlier reports (Burgett et al., 2011; Liu et al., 2014), we observed a dramatic decrease in filipin‐stained cholesterol in the plasma membrane after ICZ, but not H‐ICZ, treatment (Figure S6a). The majority of the plasma membrane cholesterol seemed to be trapped in intracellular compartments reminiscent of late endosomes and lysosomes (Liu et al., 2014; Xu et al., 2010) (Figure S6a). These experiments suggest that ICZ impedes the subcellular localization of late endosomes in the nucleoplasmic reticulum, which may explain its negative impact on EV‐mediated cell transformation.

### 3.4 ICZ‐mediated VOR complex inhibition is independent of cholesterol trafficking

**FIGURE 6:**
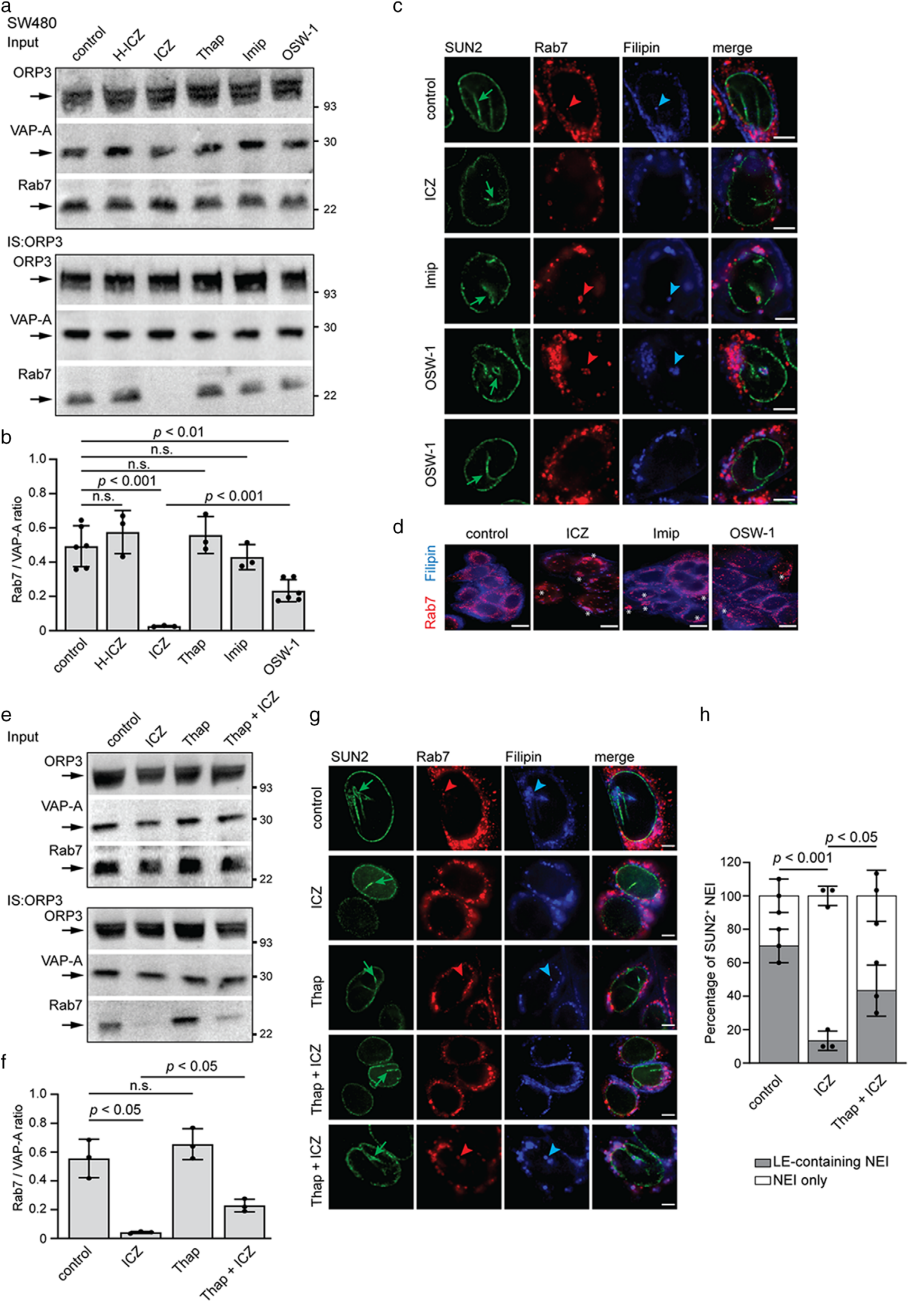
Effects of cholesterol trafficking inhibitors on the VOR complex. (a, b) Detergent cell lysates prepared from SW480 cells treated for 5 h with DMSO alone (control) or with 10 μM H‐ICZ, 10 μM ICZ, 1 μM thapsigargin (Thap), 100 μM imipramine (Imip) or 50 nM OSW‐1 were subjected to immunoisolation (IS) using anti‐ORP3 Ab followed by Protein G‐coupled magnetic beads. The input (1/50) and entire bound fractions were probed by immunoblotting for ORP3, VAP‐A and Rab7 (a). The ratio of protein immunoreactivities of the indicated pairs was quantified (b, *n* = 3–6). (c, d) Cells were incubated as in panel a prior to immunolabelling for SUN2 and Rab7 (c) or Rab7 alone (d) together with filipin staining. Cells were analysed by CLSM. Green arrows point to NEI, while red and blue arrowheads indicate Rab7^+^ late endosomes and filipin‐labelled cholesterol, respectively, within NEI. White asterisks indicate the cytoplasmic accumulation of Rab7 and cholesterol, upon drug treatments. Note that late endosomes could be found or not in NEI of OSW‐1‐treated cells. (e, f) Detergent cell lysates prepared from 6‐h mock‐treated (control) or 10 μM ICZ‐ or 1 μM Thap‐treated SW480 cells, as well as cells pre‐treated first with 1 μM Thap for 1 h followed by 10 μM ICZ for 5 h, were subjected to IS and immunoblotting as described above (a). The ratio of protein immunoreactivities of the indicated pairs was quantified (f, *n* = 3). (g) SW480 cells were incubated as in panel e prior to double immunolabelling for SUN2 and Rab7 and filipin staining. Green arrows point to NEI, while red and blue arrowheads indicate Rab7^+^ late endosomes and filipin‐labelled cholesterol, respectively, within NEI. (h) Bar graph showing the corresponding percentage of SUN2^+^ NEI containing Rab7^+^ late endosomes. For all immunoblots, molecular mass markers (kDa) are indicated, and arrows point to the proteins of interest. The mean ± S.D. are shown. N.s., not significant. Scale bars, 5 (c, g) and 10 (d) μm

To dissect the mechanism of inhibition of the VOR complex by ICZ, we studied the effect of compounds known to interfere with cholesterol distribution on the integrity of the VOR complex. For instance, an accumulation of cholesterol in late endosomes might interfere with VOR complex formation and hence with the translocation of late endosomes into nucleoplasmic reticulum. First, we exposed SW480 cells to the FDA‐approved antidepressant drug imipramine, a cationic hydrophobic amine that induces a cholesterol accumulation in late endosomes and lysosomes (Roff et al., 1991; Wichit et al., 2017; Xu et al., 2010). After exposure for 5 h to 100 μM imipramine, a non‐toxic dose that induces intracellular cholesterol accumulation (Wichit et al., 2017), cells were solubilized and ORP3 immunoisolated. Immunoblotting for ORP3, VAP‐A and Rab7 revealed that imipramine did not affect their expression and interactions (Figure 6a, b), suggesting indirectly that the activity of ICZ on the VOR complex is not directly mediated by its effect on cholesterol trafficking. In contrast, an ORPphilin molecule, OSW‐1, known to competitively inhibit 25‐HC binding to OSBP and ORP4L (Burgett et al., 2011), partially impeded the interaction of Rab7 with VAP‐A/ORP3 (Figure 6a, b). The latter data also suggest a potential role of the ORP3 ORD on ORP3‐Rab7 interaction (see below).

Next, we evaluated the impact of imipramine and OSW‐1 on the presence of Rab7^+^ late endosomes in nucleoplasmic reticulum and the general distribution of membrane cholesterol. As described above, drug‐treated cells were SUN2 and Rab7‐immunolabelled prior to filipin staining. In agreement with the co‐immunoisolation data, imipramine did not prevent the entry of Rab7^+^ late endosomes into SUN2^+^ NEI (Figure 6c). Although less drastic than with ICZ, the accumulation of membrane cholesterol in the late Rab7^+^ endosomes were observed (Figure 6d). In contrast, OSW‐1 partially inhibited the entry/presence of Rab7^+^ late endosomes in NEI consistent with the co‐immunoisolation data (Figure 6c). Analysis of lysosomal‐associated membrane protein (LAMP)‐3 (CD63) and early endosome antigen 1 (EEA1) as markers of late and early endosomes, respectively (Escola et al., 1998; Metzelaar et al., 1991; Stenmark et al., 1996), revealed the same trend for CD63 as for Rab7, whereas EEA1 is excluded from the NEI (Figure S7a, b). The latter observation is consistent with the absence of Rab5 in the NEI (Rappa et al., 2017; Santos et al., 2018). The cholesterol accumulation in late endosomes appears to be a rare event in OSW‐1‐treated cells (Figure 6d). Thus, the accumulation of cholesterol in late endosomes as observed in imipramine‐treated cells is not the primary cause of their absence in the nucleoplasmic reticulum. The same could be true for the ICZ.

Can ICZ‐induced disruption of the VOR complex and the inhibition of late endosome localization in NEI be rescued? Thapsigargin, known to increase cytosolic calcium and activate calcium‐dependent proteins required for the normal function of the endocytic pathway (Booth & Koch, 1989; Lloyd‐Evans et al., 2008), reportedly rescued the effects of ICZ on cholesterol distribution (Xu et al., 2010). The pre‐treatment with thapsigargin (1 μM) for 1 h prior to ICZ incubation rescued only partially the interaction of Rab7 with VAP‐A/ORP3 (Figure 6e, f). Thapsigargin alone did not affect the VOR complex protein interactions (Figure 6a, b, e, f). Therefore, Rab7^+^ late endosomes were found in the SUN2^+^ NEI of only a fraction of the cell population (Figure 6g, h) suggesting that, consistent with the imipramine data, the direct effect of ICZ on the VOR complex is not directly related to the general membrane trafficking of cholesterol.

### 3.5 Computer modelling and synthesis of a novel VOR complex inhibitor

**FIGURE 7:**
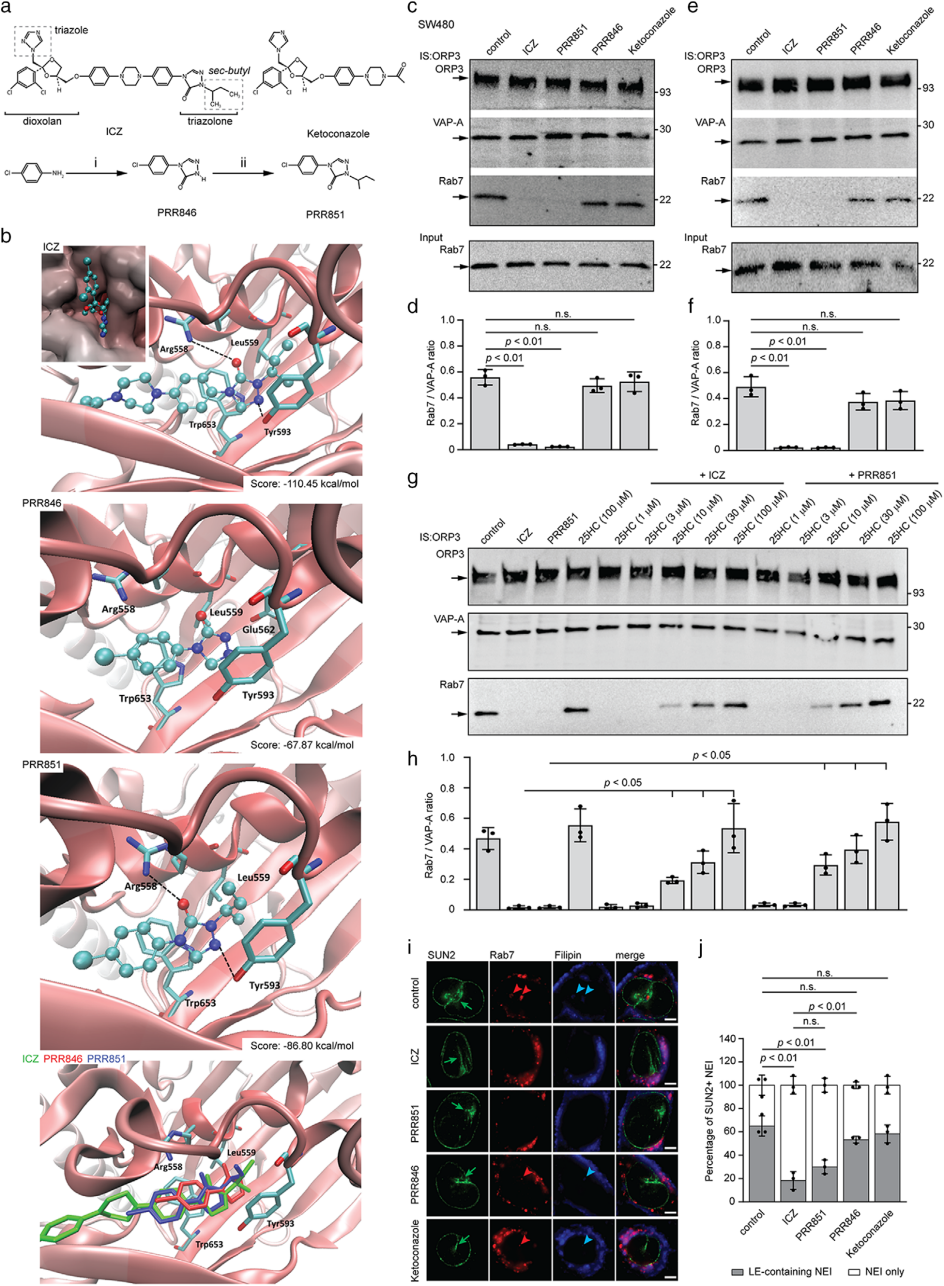
Structure of PRR846 and PRR851 compounds and the VOR complex inhibition. (a) The structures of ICZ, ketoconazole, PRR846 (4‐(4‐Chlorophenyl)‐2,4‐dihydro‐3*H*‐1,2,4‐triazol‐3‐one) and PRR851 (2‐(butan‐2‐yl)‐4‐(4‐chlorophenyl)‐2,4‐dihydro‐3*H*‐1,2,4‐triazol‐3‐one) drugs are displayed. The reactive groups in ICZ are indicated (dashed boxes). PRR846 was produced from 4‐chloroaniline treated with triethyl orthoformate *p*‐toluenesulfonic acid, methyl carbazate and sodium methoxide in methanol (reaction i), while PRR851 was generated from the N‐alkylation of PRR846 with 2‐bromobutane in the presence of sodium carbonate and 18‐crown‐6, 2‐bromobutane in DMSO (reaction ii). (b) Homology model of ORP3 ORD with the putative drug complex as computed by docking simulations for ICZ (top panel), PRR846 and PRR851 (middle panels) within the binding cavity. A composite image compares the simulations of each compound (ICZ, PRR846 and PRR851; bottom panel). For each complex, the primary ChemPLP score is indicated. The inset (top panel) is focused on the deepest portion of the ORP3 cavity and depicts the arrangement of the ICZ moieties around the cavity in the inlet. (c, d) SW480 cells were incubated with DMSO (control), 10 μM ICZ, PRR851, PRR846 or ketoconazole for 5 h, solubilized and subjected to immunoisolation (IS) using anti‐ORP3 Ab followed by Protein G‐coupled magnetic beads. The input (1/50, for Rab7 only) and entire bound fractions were probed by immunoblotting for ORP3, VAP‐A and Rab7 (c). The ratio of protein immunoreactivities of the indicated pairs was quantified (d, *n* = 3). (e‐h) Detergent SW480 cell lysates were incubated with DMSO (control), 10 μM ICZ, PRR851, PRR846 or ketoconazole for 30 min on ice (e), and then subjected to IS and immunoblotting as described above. Alternatively, lysates were pre‐incubated with 25‐HC at the indicated concentration for 30 min prior to drug treatments (g). As internal controls, cell lysates were treated for 1 h with DMSO (control), 10 μM ICZ, 10 μM PRR851 or 100 μM 25‐HC. The ratio of protein immunoreactivities of the indicated pairs was quantified (f, h, *n* = 3). The molecular mass markers (kDa) are indicated, and arrows point to the proteins of interest (c, e, g). (i, j) SW480 cells were incubated with DMSO (control), 10 μM ICZ, PRR851, PRR846 or ketoconazole for 5 h prior to double immunolabelling for Rab7 and SUN2 and staining with filipin. Cells were analysed by CLSM (i). Arrow indicates SUN2^+^ NEI (green) and arrowheads point to Rab7 (red) and filipin‐labelled cholesterol (cyan) therein. Bar graph shows the percentage of SUN2^+^ NEI containing Rab7^+^ late endosomes (LE) (j). The mean ± S.D. are shown. *P* values are indicated. N.s., not significant. Scale bars, 5 μm

ICZ, a traditional triazole antifungal drug, has been reported to inhibit enterovirus and hepatitis C virus replication by targeting OSBP and its homologue ORP4, indicating various molecular targets. Since our goal was to design specific compounds able to inhibit the VOR complex and consequently the nuclear transfer of EV components, we simulated the interaction of ICZ with ORP3 ORD (Figure 7a). We used a homology model of ORP3 based on crystallographic data recently reported for cholesterol‐bound OSBP‐related protein 1 (ORP1) complex (see Materials and Methods) (Dong et al., 2019). The computed complex between the modelled ORP3 ORD and the cis (2R, 4S, 2′R)‐stereoisomer of ICZ is presented, focusing on its 4‐phenyl‐2‐butan‐2‐yl‐1,2,4‐triazol‐3‐one portion, that is, inserted into the deepest region of the cavity, while the 2,4‐dichlorophenyl‐2‐(1,2,4‐triazol‐1‐ylmethyl)‐1,3‐dioxolane substructure remains outside or at most on the rim of the cavity (Figure 7b, see inset in top panel). In detail, the 1,2,4‐triazol‐3‐one group is engaged by a reinforced H‐bond between its carbonyl oxygen atom and residue Arg_558_, which additionally stabilizes charge transfer interactions with the 4‐phenyl ring (Figure 7b, top panel). The 1,2,4‐triazol‐3‐one ring approaches Tyr_593_ as well, which elicits both H‐bonds plus π−π stacking interactions. Furthermore, the 4‐phenyl ring is engaged in a π−π stacking with Trp_653_, while the 2‐alkyl chain stabilizes hydrophobic contacts with Leu_559_. By contrast, the 2,4‐dichlorophenyl‐2‐(1,2,4‐triazol‐1‐ylmethyl)‐1,3‐dioxolane portion is seen to elicit a markedly more limited number of interactions. Rationalizing the described interactions, the key contacts appear to be those involving the 4‐phenyl‐2‐butan‐2‐yl‐1,2,4‐triazol‐3‐one portion with the deepest residues, such as Arg_558_, Tyr_593_ and Trp_653_, while the remaining part of the molecule is involved in rather weak hydrophobic bonds, especially considering that the potential polar contacts stabilized by the most external moieties (such as those involving Arg_698_) should be partly shielded by the surrounding solvent molecules. On these grounds, the differences of activity between the ICZ stereoisomers (Bauer et al., 2018) could be mostly ascribed to the greater steric hindrance of the trans stereoisomers, which would prevent a proper insertion of the 4‐phenyl‐2‐butan‐2‐yl‐1,2,4‐triazol‐3‐one system within the deepest cavity region rather than different interactions stabilized by the 2,4‐dichlorophenyl‐2‐(1,2,4‐triazol‐1‐yl‐methyl)‐1,3‐dioxolane portion on the cavity rim. The pivotal role of these is indirectly confirmed by the inactivity of H‐ICZ. Among the 10 generated poses, only in one case was H‐ICZ able to insert 4‐phenyl‐2‐butan‐2‐yl‐1,2,4‐triazol‐3‐one into the deepest region of the ORP3 cavity; however, it was unable to assume the optimal arrangement as ICZ (Figure 7b, top panel), but rather a more superficial arrangement, missing the key contacts with Arg_558_, Tyr_593_, and Trp_653_ (data not shown).

Based on the computed data, we synthesized a series of related compounds smaller than the lead ICZ with fewer functional groups, notably missing the dioxolan region that carries a dichlorophenyl ring and the triazole moiety, thus reducing the possibility of side effects unrelated to the VOR complex inhibition (Figure 7a). Among them, PRR851, that bears the same 2‐butanyl side chain bound to the nitrogen in position 2 of the triazolone ring of ICZ, was assessed, while PRR846, the related unsubstituted derivative at the same position devoid of side chain, was used as a potential negative control (Figure 7a). The key role of the above‐mentioned residues was confirmed by the docking results for the two proposed derivatives. The lack of the alkyl chain prevented the proper arrangement of PRR846, which in all generated views appeared unable to stabilize the key contacts with Arg_558_, Tyr_593_ and Trp_653_ (Figure 7b). Thus, PRR846 assumed a deeper position compared to the corresponding ICZ portion by which the triazole ring contacts Glu_562_ while missing the key interactions. In sharp contrast, both PRR851 enantiomers appeared comparable with the corresponding ICZ moiety. In the figure 6b, (*R*)‐PRR851 is displayed, since it shows a slightly better score compared to (*S*)‐PRR851. It assumed a configuration very similar to that exhibited by the 4‐phenyl‐2‐butan‐2‐yl‐1,2,4‐triazol‐3‐one system of ICZ, and elicited a rather superimposable set of interactions comprising both polar and hydrophobic contacts. Moreover, the satisfactory arrangement of (*R*)‐PRR851 was seen with marginal differences in seven out of 10 computed poses while a greater heterogeneity was observed for PRR846 complexes, in which the ligand assumed superficial and/or flipped arrangements, but never assumed a convenient fit. Finally, the optimal arrangements of (*R*)‐PRR851, PRR846 and ICZ within the ORP3 ORD are shown simultaneously (Figure 7b, bottom panel). This confirms the superimposable configuration of (*R*)‐PRR851 with corresponding moieties of ICZ, while PRR846 showed a deeper arrangement whereby the 1,2,4‐triazol‐3‐one ring took the place of the alkyl chain, missing key contacts with the ORP3 residues.

### 3.6 PRR851 interferes with the VOR complex integrity

To evaluate experimentally the impact of PRR compounds on the VOR complex, SW480 cells were treated for 5 h with 10 μM PRR851 or PRR846, a concentration within the range of clinically attainable ICZ serum concentrations at steady‐state. For comparison, we employed 10 μM ICZ or ketoconazole, an imidazole that acts like ICZ on the lanosterol 14α‐demethylase enzyme via the triazole moiety (Strushkevich et al., 2010) (Figure 7a). Afterward, cells were solubilized and ORP3 was immunoisolated. Recovered materials were probed for ORP3, VAP‐A and Rab7 by immunoblotting. The co‐immunoisolation of Rab7 was selectively altered in cells treated with PRR851, but not with PRR846 (Figure 7c). The Rab7/VAP‐A ratios were similar in samples treated with PRR851 and ICZ (Figure 7d). The inhibition of the protein interactions was also observed at lower PRR851 concentration (5 μM; Figure S8a, b). In contrast, ketoconazole did not influence the VAP‐A/ORP3/Rab7 interactions. Similar data were obtained when the new drugs were incubated with detergent cell lysates instead of living cells (Figure 7e, f).

To investigate whether ICZ and PRR851 acted on the highly conserved ORP3 ORD, whose hydrophobic pocket binds a single sterol, we evaluated the potential competition of 25‐HC to ICZ or PRR851‐mediated VOR complex disruption. We pre‐incubated the detergent SW480 cell lysates with 25‐HC at various concentrations for 30 min prior to ICZ or PRR851 treatments. Interestingly, we observed that 25‐HC inhibits in a dose‐dependent manner the action of both drugs, suggesting that they act on ORP3 ORD (Figure 7g, h) in agreement with the OSW‐1 data (Figure 6).

To visualize the effect of drugs on the transport of late endosomes to the nucleoplasmic reticulum, the distribution of Rab7 and cholesterol was investigated as described above. Similar to ICZ‐treated cells, Rab7 and cholesterol were reduced in NEI of cells exposed to PRR851 (Figure 7i). A decrease of about 30% and 40% of SUN2^+^ NEI harbouring Rab7^+^ late endosomes upon PRR851‐ and ICZ‐treatment, respectively, was observed (Figure 7j). Neither PRR846 nor ketoconazole affected the presence of Rab7 or cholesterol in NEI (Figure 7i, j). Differently from ICZ‐treated cells, the general redistribution of cholesterol, particularly between endocytic organelles and the plasma membrane, was not evidenced upon PRR851‐ or PRR846‐treatment (Figure S6b). Consistent with an earlier study (Strating et al., 2015), a modest redistribution of cholesterol was observed in ketoconazole‐treated cells, including accumulation of Rab7^+^ late endosomes (Figure S6b). These data suggest that the effects observed with PRR851 were due to the disruption of the VOR complex and not on the general cholesterol distribution.

**FIGURE 8:**
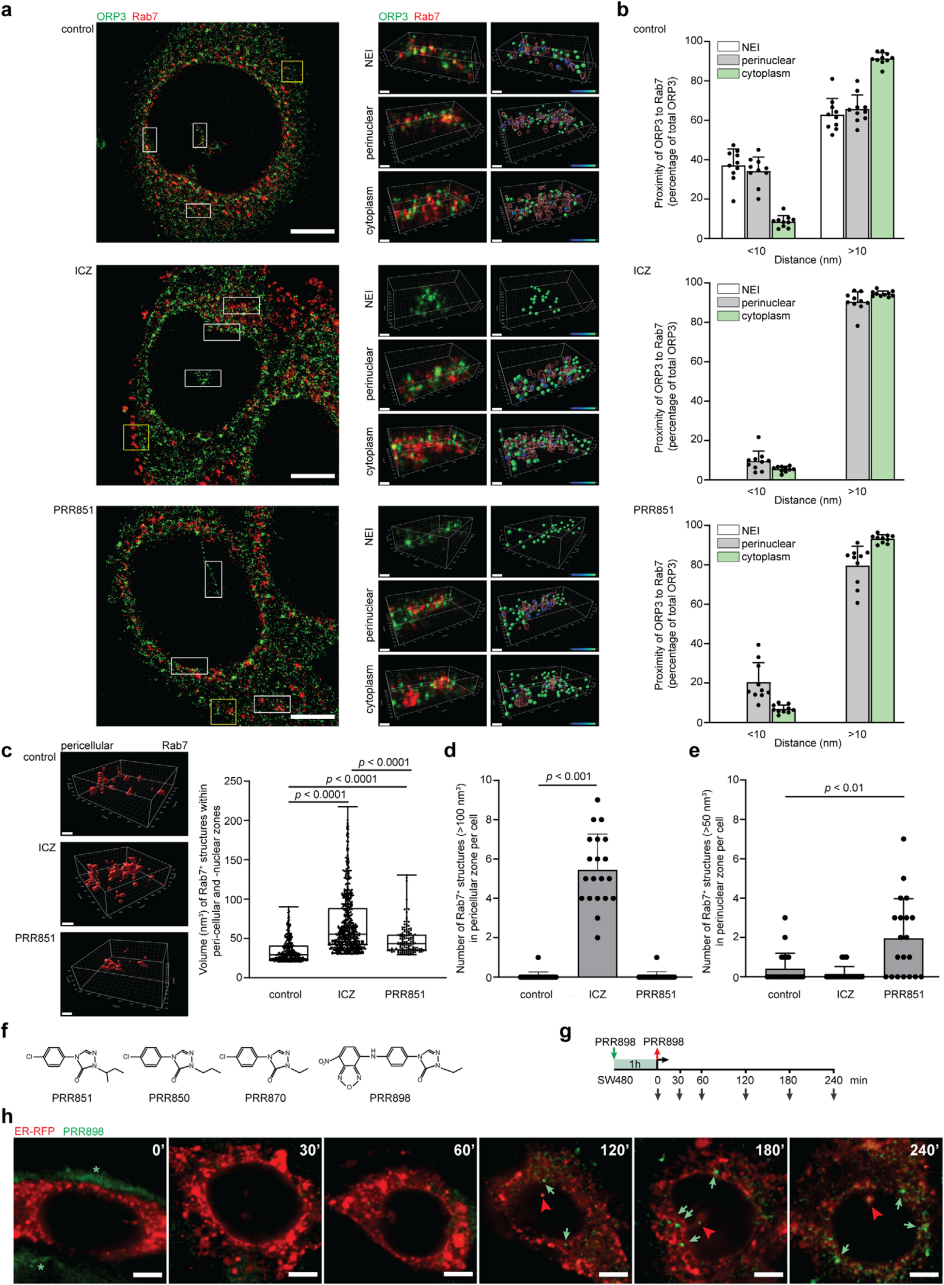
Impact of ICZ and PRR851 on the proximity of ORP3 and Rab7 in various subcellular zones. (a‐e) SW480 cells were treated with 10 μM ICZ or PRR851 or with the DMSO (control) for 5 h, fixed and the subcellular localization of ORP3 (green) and Rab7 (red) was analysed by immunolabelling using 3D dSTORM as described under ‘Materials and Methods’. Whole cell images (a, left panels) and magnified areas (white boxes) of NEI, perinuclear and cytoplasm (a, right panels) are displayed. Corresponding Imaris‐based, 3D‐rendered images were reconstructed and a computer model was rendered to show the relative structure and distance between ORP3 and Rab7 with the heat map indicating their linear proximity (blue to green: 0 to 50 nm) (a). The distance between the total ORP3 molecules and Rab7 in each subcellular area was scored as inferior or superior to 10 nm and data plotted as percentage of total ORP3 in each cell (*n* = 10 independent cells from three distinct experiments) (b). 3D‐rendered images from pericellular zone as indicated in panel a (yellow box) were reconstructed to illustrate the difference in volume of Rab7^+^ structures of control compared with ICZ‐ and PRR851‐treated cells (c). The volume of Rab7^+^ structures greater than 20 nm^3^ in the pericellular and perinuclear zones is depicted (*n* = 20 independent cells were analysed per condition) (c). The numbers of Rab7^+^ structures with a volume greater than 100 or 50 nm^3^ in the pericellular and perinuclear zone, respectively, of a given cell are graphed (*n* = 20 cells) (d, e). (f) Chemical structures of PRR analogues including the fluorescent compound PRR898. (g) Methodology of the PRR898 experiments. (h) SW480 cells expressing ER‐RFP (red) were exposed to 2 μM PRR898 (green) for 1 h, washed and incubated for the indicated time prior to CLSM analysis. Note the progressive accumulation of PRR898 in the perinuclear zone as indicated by an increase in fluorescence intensity (green arrow). The asterisk indicates the fluorescence of PRR898 outside the cell after 1‐h incubation, while the red arrowhead indicates PRR898 inside the NEI. The mean ± S.D. and *P* values are presented. Scale bars, 5 μm (a, left panels; h), 500 nm (a, right panels; c, top and middle panels), 400 nm (c, bottom panel)

To get more insight about the VOR complex protein interactions, we employed three‐dimensional (3D) direct stochastic optical reconstruction microscopy (dSTORM), a single molecule super‐resolution technique. The co‐localization of ORP3 and Rab7 was evaluated by immunolabelling since the binding of the latter required the interaction of ORP3 and VAP‐A. dSTORM data confirmed the absence of Rab7 in NEI in cells treated with ICZ and PRR851 (Figure 8a, left panels). The 3D‐rendered images of three specific cellular regions, that is, NEI, perinuclear zone and cytoplasm, were reconstructed and a computer model was rendered to evaluate the linear proximity between ORP3 and Rab7 (Figure 8a, right panels). In the control samples, about 35% of the total ORP3 observed in the NEI and perinuclear zones were at a short distance (< 10 nm) from Rab7 (Figure 8b, upper graph). In contrast, ORP3‐Rab7 pairs were rarely detected in the cytoplasm, although the vast majority of ORP3 and Rab7 molecules were found there. In addition to the negative effect on the localization of Rab7 in NEI, ICZ reduced the proximity between ORP3 and Rab7 in the perinuclear zone (Figure 8b, middle graph). A similar observation was made with PRR851 (Figure 8b, bottom graph). The influence of both drugs on cytoplasmic ORP3‐Rab7 pairs was marginal due to the limited amount of these interactions outside the nuclear regions. In addition to the impact of drugs on the ORP3‐Rab7 proximity, Rab7^+^ structures were significantly increased in volume, especially in ICZ‐treated cells, where large structures greater than 100 nm^3^ were observed (Figure 8c). An accumulation of Rab7^+^ large endosomes/vacuoles (> 100 nm^3^) in pericellular zone in ICZ‐treated cells (Figure 8d) and smaller ones (> 50 nm^3^) in perinuclear zone in PRR851‐treated cells were detected (Figure 8e). The two last observations suggest that ICZ‐mediated cholesterol accumulation in late endosomes affects partially their trafficking from pericellular to perinuclear zones, a situation that did not occur with PRR851, in line with the filipin data. These observations are also in agreement with the reduced velocity of late endosomes in the presence of ICZ (see above). The accumulation of late endosomes in perinuclear areas and their absence in NEI upon PRR851 treatment suggest that the interaction between Rab7 and ORP3‐VAP‐A complexes occurs at the perinuclear region *en route* to NEI.

To visualize the interaction of PRR compounds with ORP3–VAP‐A complex, we designed a fluorescent analogue, named PRR898, containing a 7‐nitrobenzo [1,2,5] oxadiazole moiety (Figure 8f). We used a version of PRR851 with a shorter alkyl side chain. The lack of one or two methyl groups observed in PRR850 and PRR870, respectively (Figure 8f), did not prevent their potential interaction with the ORD domain of ORP3 (computer modelling not shown), as illustrated experimentally by the lack of Rab7 in ORP3‐driven co‐immunoisolation performed on drug‐treated cells or detergent lysates (data not shown). The same observation was made with fluorescent PRR898 indicating that it inhibits the binding of Rab7 to ORP3/VAP‐A complex. SW480 cells expressing ER‐RFP were then incubated with PRR898 (2 μM) for 1 h and observed immediately or after 30, 60, 120, 180 and 240 min of chase by CLSM (Figure 8g). In the first 60 min, PRR898 appeared as a weak and discrete signal distributed throughout the cytoplasm (Figure 8h); bright signals began to develop particularly in the perinuclear zone, especially at the entrance of the NEI, as observed after 180 min, suggesting an accumulation of the drug where ORP3–VAP‐A complexes possibly occur.

### 3.7 PRR851 inhibits pro‐metastatic morphological transformation

**FIGURE 9:**
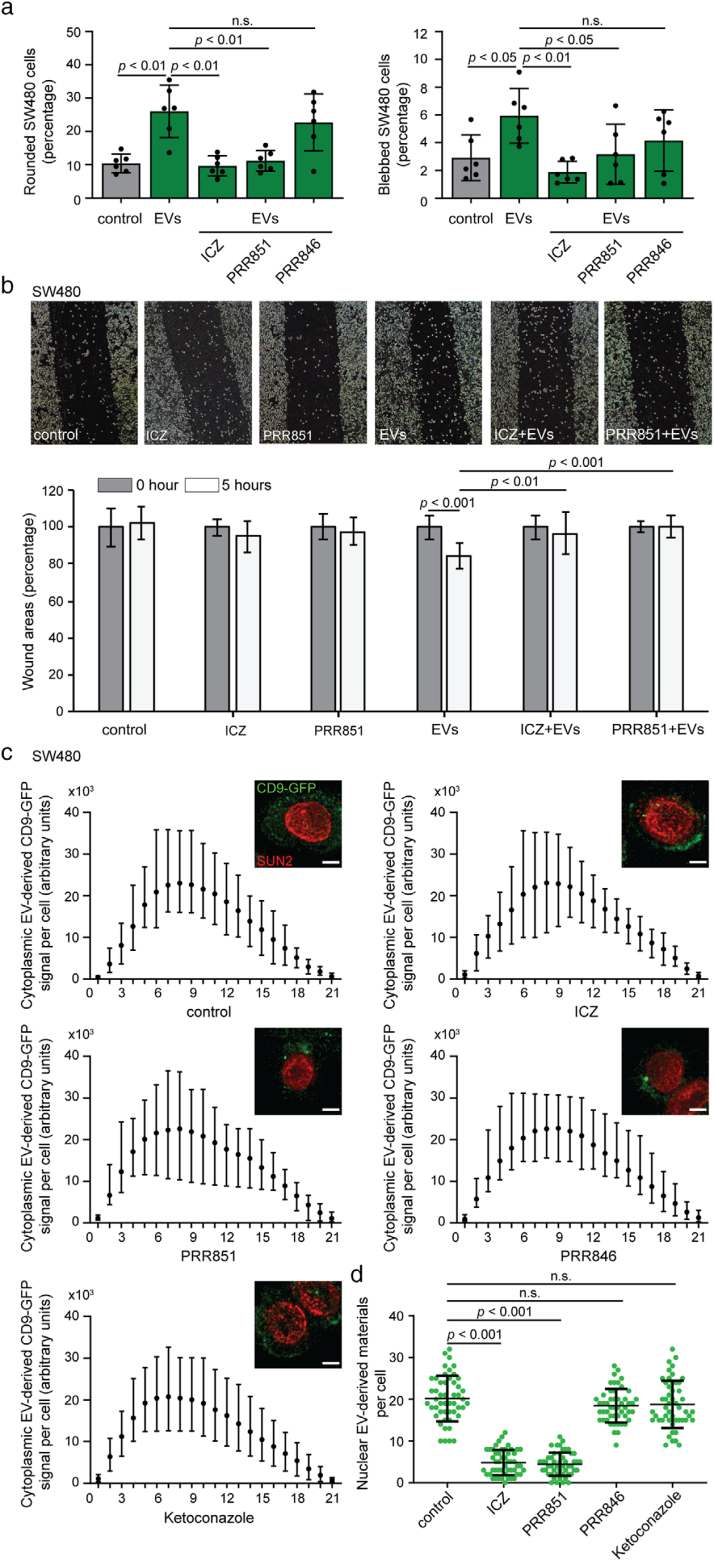
PRR851 inhibits EV‐induced pro‐metastatic morphological transformation and nuclear transfer of EV cargo in SW480 cells. (a) SW480 cells were pre‐treated with DMSO (control), 10 μM ICZ, PRR851 or PRR846 for 10 min prior to incubation without or with EVs (1 × 10^9^ particles/ml) derived from SW620 cells for 5 h prior to CD9 immunolabelling. Cell morphology was analysed by CLSM. Bar graphs show the percentage of cells harbouring a rounded (left) or blebbed (right) morphology upon exposure to EVs. The mean ± S.D. are shown. At least 50 cells were evaluated per condition and independent experiment (*n* = 6). (b) Representative images of the scratch wound healing assay of SW480 cells treated with 10 μM drugs (ICZ, PRR851) without or with EVs (7.5 × 10^8^ particles/ml) derived from SW620 cells for 5 h (upper panels). The percentage of the remaining wound area was assessed as described in Materials and Methods. The mean ± S.E.M. (*n* = 2 independent experiments) is presented (lower panel). (c, d) SW480 cells were pre‐treated for 10 min with DMSO (control), 10 μM ICZ, PRR851, PRR846 or ketoconazole prior to 5‐h incubation with FEMX‐I cell‐derived CD9‐GFP^+^ EVs in the absence or presence of drugs. Cells were then fixed and immunolabelled for SUN2. The amounts of EV‐derived CD9‐GFP in the cytoplasmic (c) and nuclear (d) compartments were analysed by CLSM and quantified. Mean with the range of green fluorescence per slice from 10 individual representative cells are displayed as well as composites of 21 optical x‐y sections (c, inset). Nuclear CD9‐GFP was evaluated from 50 cells per condition and a representative experiment with mean ± S.D. is shown (d). *P* values are indicated. N.s., not significant. Scale bars, 5 μm

We evaluated whether PRR851, like ICZ, could inhibit EV‐mediated cell morphological transformation as well as cell motility. SW480 cells were pre‐treated with 10 μM PRR851 or PRR846 as well as ICZ as a positive control for 10 min prior to incubation for 5 h with EVs (1 × 10^9^ particles/ml) derived from SW620 cells. Morphological alterations were then assessed after cell fixation and immunolabelling of CD9 to visualize the plasma membrane and its blebs (Figure 9a). The number of rounded cells or those with membrane blebs were significantly lower in PRR851‐ and ICZ‐treated cells compared to mock‐treated cells exposed to EVs (Figure 9a). Similar to the controls, no effect was observed with PRR846, suggesting that this chemical drug did not prevent the EV‐mediated cell morphological transformation (Figure 9a). Similar data were obtained when 2 × 10^9^ particles/ml were used (Figure S9). In the latter case, doubling the amount of EVs increased the proportion of rounded recipient cells and their bleeding. To complement these experiments, we assessed cell motility. To this end, a scratch wound healing assay was performed where SW480 cells were treated with drugs (ICZ or PRR851) in the absence or presence of SW620 cell‐derived EVs for 5 h. Remarkably, both drugs reduced the recovery of wound areas, that is, stimulated by EVs (Figure 9b).

These data prompted us to visualize the impact of drugs on endocytosis and nuclear transfer of EV components using CD9‐GFP^+^ EVs as described above. After 5 h of incubation of CD9‐GFP^+^ EVs with drug‐treated cells, none of the chemicals interfered with EV endocytosis, as shown by the intensity of CD9‐GFP cytoplasmic signals (Figure 9c). Similar data were obtained using membrane dye‐labelled EVs and flow cytometry analysis (data not shown). In contrast, the nuclear transfer of CD9‐GFP was significantly reduced in PRR851‐treated cells as reported above for ICZ, but not in cells treated with PRR846 or ketoconazole (Figure 9d; Figure S3d). Quantification of three independent experiments revealed that nuclear GFP in PRR851‐treated cells was reduced to 4.1 ± 0.9 (mean ± S.D.) punctate signals per nucleus similar to ICZ‐treated cells, that is, 4.6 ± 0.4, while control cells and PRR846‐ or ketoconazole‐treated cells had values of 19.9 ± 2.2, 18.1 ± 1.7 and 18.5 ± 1.0, respectively. The difference between PRR851‐ or ICZ‐treated cells and control cells was statistically significant (*P* values of 0.005 and 0.01, respectively). Altogether, these data suggest that the impact of drugs on morphology and migration of colon cancer cells depends on the integrity of the VOR complex and consequently on the nuclear transfer of EV cargo.

### 3.8 Toxicity of ICZ and PRR851

Finally, the cytotoxicity of the drugs was evaluated using colorimetric assay (MTS reagent) as described in Materials and Methods. Cell growth was assessed every 24 h for a total of 72 h. SW480 and FEMX‐I cells were used as well as primary human MSCs and fibroblasts, the latter often being the first targets for EVs derived from cancer cells in the tumour microenvironment. ICZ, regardless of its concentrations, had little effect on the growth of SW480 cells, whereas H‐ICZ caused a dose‐dependent delay in cell growth (Figure S10a). After 72 h of incubation, 10 μM ICZ and H‐ICZ significantly reduced the number of metabolically active FEMX‐I cells (Figure S10b). Both drugs appeared to reduce the growth of MSCs, especially at higher concentrations (Figure S10c). In contrast, only H‐ICZ had an inhibitory effect on fibroblasts (Figure S10d). We then investigated the effects of PRR compounds and ketoconazole (Figure S10e, f). At 10 μM, PRR851 and PRR846 exposure had little effect on cancer cells and MSCs over 72 h. Thus, their effect on cell growth was milder than ICZ, particularly on primary cells. Ketoconazole had no effect on cancer cells but reduced the growth of MSC similarly to ICZ.

## 4 DISCUSSION

By studying EV‐cell communication using biochemical, loss‐of‐function and high‐resolution microscopy techniques, we have demonstrated that FDA‐approved ICZ and a novel small compound PRR851 interfere with EV‐mediated pro‐metastatic morphological transformation and migration behaviour of cancer cells by acting as inhibitors of nuclear transfer of late endosome‐carried EV cargo. Silencing ORP3 or VAP‐A has been shown to block the nuclear transfer of EV cargo in melanoma and HeLa cells (Santos et al., 2018) as well as cell transformation of colon cancer cells (this study). Both drugs can recapitulate these cellular effects. Along with these inhibitions, they interfered with the integrity of the VOR complex and the entry of late endosomes into the nucleoplasmic reticulum, suggesting that the latter are responsible for the above‐mentioned effects. Knowing that cancer cells hijack their surrounding microenvironment and that EVs derived from them determine the pre‐metastatic niche, inhibitors of nuclear transfer of EV cargo into host cells could find cancer therapeutic application, notably in combination with direct targeting of cancer cells. Our data on SW480 cell transformation by SW620 cell‐derived EVs and its inhibition by ICZ/PRR851 illustrate this potential use.

Repurposing of the antifungal agent ICZ for cancer has been based on the identification of several potential cancer targets, including the Hedgehog and the mTOR pathways (Head et al., 2015; Kim et al., 2010), but not on its action on proteins of the OSBP family. Compared to ICZ, PRR851 has many advantages and fewer negative effects on cell growth or other cellular events, making it an important breakthrough in the search for an inhibitor of intercellular communication. For instance, the molar concentrations of ICZ and PRR851 required for inhibition of the VOR complex were similar; however, due to the ∼3‐fold lower molecular weight of PRR851, the absolute amount of drug required to achieve the same effect was considerably less. The lack of moieties that inhibit other non VOR complex‐related cellular targets, suggests that PRR851 should have a clinically more favourable toxicity profile, which is consistent with MTS assay. Moreover, PRR851 has only one chiral centre (in contrast to three in ICZ), which is present a simpler situation, both in terms of stereoselective synthesis and potency of the two stereoisomers. Importantly, molecular docking indicated that both possible stereoisomers have similar positioning in the ORD pocket.

The scheme utilized for the synthesis of PRR851 can be easily modified to incorporate side chains of variable lengths as illustrated with PRR850, PRR870 and fluorescent PRR898 analogues. The latter aspect is interesting since its structure can be adapted to interfere with the function of other OSBP‐related proteins, where subtle conformational changes can be observed in the hydrophobic pockets of their ORD domain (Olkkonen, 2015). The sequence and modelling analyses of ORD domain of various ORP proteins (i.e., OSBP, ORP1, ORP2, ORP4, ORP6, ORP7 and ORP9) including the key residues Arg_558_, Tyr_593_ and Trp_653_ that play an essential role in stabilizing the ORP3–PRR851 complex, suggest that the key triad is present and/or in a configuration accessible to PRR851 inhibition only in ORP7 in addition to ORP3 (Figure S11a; data not shown). This may also explain the apparently limited toxicity of PRR851. The potential impact of PRR851 to other OSBP‐related proteins, notably ORP7, needs to be investigated as well as their subcellular localization. Until now, only ORP3, but not OSBP and ORP1L, was reported in the NEI (Santos et al., 2018).

The absence of major *in vivo* effects on the cellular redistribution of cholesterol and on the translocation of late endosomes from the cytoplasmic to the perinuclear zone by PRR851, in comparison to ICZ, are important issues as is the lack of antifungal activity, since the main targets, besides cancer cells, are physiologically normal cells such as MSCs, which can be transformed by cancer EVs. The imipramine data suggest that the cholesterol accumulation in the endosomal compartment and/or lysosomes per se as observed after the ICZ treatment is not necessarily the main cause of VOR complex disruption. The latter is also supported by *in vitro* data performed with detergent cell lysates. Nonetheless, it remains to be determined whether PRR851 could bind, albeit in a milder manner, to the sterol‐sensing domain of regulatory sterol‐carrier proteins, or regulate their expression, as has been shown for ICZ (Liu et al., 2014; Trinh et al., 2017). The next step will be to determine the molecular motors and effectors allowing the translocation of late endosomes into NEI, a step that appears to be dependent of a microtubule network (Santos et al., 2018). Therein, a Rab7 effector such as Rab7‐interacting lysosomal protein may play a role in the regulation of late endocytic traffic as reported in the cytoplasmic compartment (Cantalupo et al., 2001). The accumulation of enlarged Rab7^+^ structures in the vicinity of the nuclear membrane in cells treated with PRR851 or at the cellular periphery of those treated with ICZ suggests that the movement of late endosomes (or effector regulating it) in the NEI and cytoplasm may be distinct. For the moment, it cannot be excluded that a certain threshold of cholesterol content within the membrane of the late endosome influences their translocation (Olkkonen, 2015; Rocha et al., 2009). An involvement of the components of the nuclear pores that could facilitate the docking of the late endosomes with the nuclear membrane is not excluded. The accumulation of fluorescent PRR analogue at the perinuclear zone, especially at the entrance of the NEI is consistent with it. Finally, it should be mentioned that only the phosphorylated form of ORP3 seems to be involved in VAP‐A/Rab7 interactions. Mechanically, the binding of ICZ/PRR851 to the ORD domain of phosphorylated ORP3, which is engaged in a VAP‐A interaction notably at perinuclear zones, could lead to an ORP3 conformational change that would impede the recognition of Rab7 and late endosomal membrane (Figure S11b). Inter‐relationships of the functional domains of ORP3 in membrane targeting have been demonstrated (Lehto et al., 2005; Olkkonen, 2015; Weber‐Boyvat et al., 2015).

In conclusion, we described that disruption of the VOR complex with chemical compounds interfered with the transport of EV cargo into the nuclear compartment, resulting in the inhibition of the pro‐metastatic morphological transformation of cancer cells and their migratory properties. Applications in the field of cancer, and possibly in virology, where late endosomal compartments are involved, could be developed.

## CONFLICTS OF INTEREST

The United Kingdom patent applications GB1810556,9 and GB2014012.5 and United States provisional patent number US62/690,616 are pending. The authors declare no other competing interests.

## AUTHOR CONTRIBUTIONS

Designed the experimental approach, performed the experimental work and analysed the data, Mark F. Santos, Germana Rappa, Jana Karbanová, Simona Fontana, Maria Antonietta Di Bella, Marta Moschetti and Riccardo Alessandro; Performed the experimental work and analysed the data, Gyunghwi Woo, Kevin Huang and Tony Huynh; Performed mass spectrometry and conducted proteomic data analysis, Marshall R. Pope; Designed and synthetized the chemical drugs, Barbara Parrino, Stella Maria Cascioferro, Patrizia Diana and Girolamo Cirrincione; Performed the molecular modelling and analysed the data, Giulio Vistoli; Discussed the hypothesis and contributed to data interpretation and manuscript writing, Germana Rappa and Goffredo O. Arena; Conceived the hypothesis, designed the experimental approach, proposed and designed the chemical drugs, analysed and interpreted the data, coordinated the project, wrote the first draft of the manuscript and finalized it, Denis Corbeil and Aurelio Lorico; Discussed the hypothesis and contributed to manuscript reading, providing feedback and editing the final version, all authors.

## Supporting information

Supporting Information

Supporting Information

Supporting Information

Supporting Information

Supporting Information

Supporting Information

Supporting Information

Supporting Information

Supporting Information

Supporting Information

## Data Availability

The datasets used and/or analysed during the current study are available from the corresponding author on reasonable request.
