# Supplementary material for: Itraconazole inhibits nuclear delivery of extracellular vesicle cargo by disrupting the entry of late endosomes into the nucleoplasmic reticulum
Source: J Extracell Vesicles. 2021 Aug 14;10(10):e12132. doi: 10.1002/jev2.12132 (PMC8363911; doi:10.1002/jev2.12132)
Supplement: Supplementary file 1 — Supporting Information [file JEV2-10-e12132-s006.pdf]

Supporting Information

Supplementary Materials and Methods

Mass spectrometry

TMT reagent	126	127N	127C	128N	128C	129N	129C	130N	130C	131
Sample	1x HeLa	VAPA_1a	VAPA_1b	Orp3_1a	Orp3_1b	VAPA_2a	VAPA_2b	Orp3_2a	Orp3_2b	4x HeLa
Inject_1: F1	S1	S3	S4	S5	S6	S7	S8	S9	S10	S11
Inject_2: F2	S2	S12	S13	S14	S15	S16	S17	S18	S19	S20

Scheme for TMT 10-plex labelling (see above). As described in Material and Methods, alternate target affinity enrichments for VAPA and ORP3 were conducted in duplicate. In the row labelled ‘sample’ either biological replicate is indicated by ‘_1’ or ‘_2’ following the target compound. One quarter of recovered proteins were split into 2 equal partitions which are labelled ‘a’ and ‘b’ respectively. Hence the 8 TMT reagents 127 – 130 provide the means to track proteins recovered for each VAPA and ORP3 targeted pull-down in 2 biological replicates (BR). For each BR, the labels tag two technical replicates (TR) as well. The two remaining TMT label reagents (126 and 131) were used to tag a standard HeLa cell digest (Thermo Fisher Scientific) which, added to the PD in a 1:4 ratio, provided to 20 ng of 126 label and 100 ng of 131 in each 4 μL injection.

The Table S5 (Excel file) summarizes evidence for the presence and relative abundance of three proteins identified with untargeted DDA LC-MS/MS and Sequest searching using Proteome Discoverer ver. 2.4 (Thermo Fisher Scientific, Waltham MA). Acquisition and Search parameters are described in the main Methods. Among the quality controls coded into the analysis are restrictions on reporter ion abundance and isolated ion purity. The first restricts total reporter ion signal to noise (S/N) to events that sum to at least 100 across all channels. In

addition, the analysis method rejects spectral matches if the ion isolation event records greater than 50% interference from ions other than the precursor. Such blank records are labelled “Excluded by the method”.

The worksheet information is arranged in three tiers of layered categories representing protein (blue), peptide (orange) and spectral matches (grey). Relative to the headings of each category, the individual records are lighter in color. With the information (Table S5), the titles of each column are self-explanatory except for abundance.

In the third-tier category (spectral matches, grey) ‘abundance’ refers to the raw signal to noise (S/N) of ion current detected in the Orbitrap for each reporter ion mass channel. At the peptide level, the raw abundances are aggregated and scaled such that the total ‘Grouped Abundance’ is equal to 100x the number of reporter ion channels. That is, for the TMT 10-plex, the sum of ‘grouped abundances’ is 1000 per injection on the LC-MS. Grouped abundances at the protein level are a weighted average across sibling peptides according to the Protein Prophet algorithm (Nesvizhskii et al., 2003). Again, these are also normalized so that the total for all reporter channels is 1000.

Nesvizhskii, A. I., Keller, A., Kolker, E. and Aebersold, R. A. (2003). Statistical model for identifying proteins by tandem mass spectrometry. *Anal Chem, 75*, 4646-4658.

Supplementary Figures

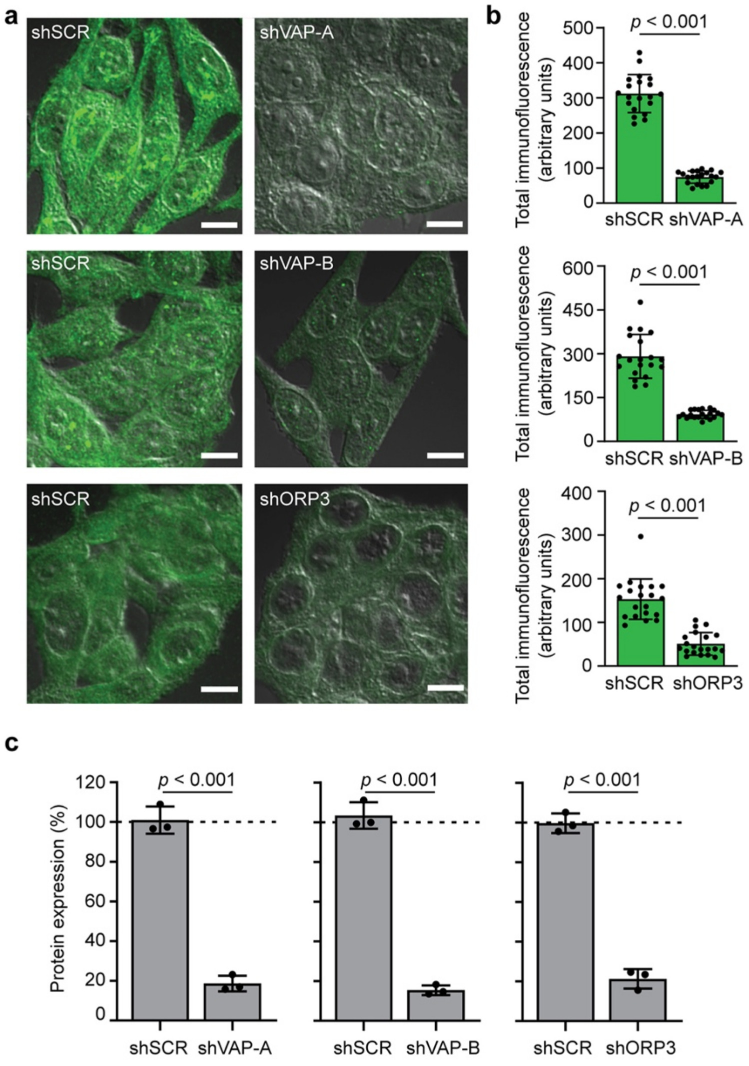

FIGURE S1 Knockdown of ORP3, VAP-A and VAP-B in SW480 cells. (a, b) SW480 cells stably transfected with plasmids carrying scrambled shRNA (shSCR), shORP3, shVAP-A or shVAP-B were immunolabelled for VAP-A, VAP-B and ORP3, and analyzed by CLSM (a). The immunofluorescence was quantified using Fiji (b, n = 20 cells). (c) Cells were analyzed by immunoblotting for VAP-A, VAP-B , ORP3 and β-actin. The relative expression of VAP-A, VAP-B and ORP3 was quantified by comparison with the untransfected control (dotted line). The samples were normalized to β-actin (n = 3). The mean ± S.D. are shown. *P* values are indicated. Scale bars, 10 μm.

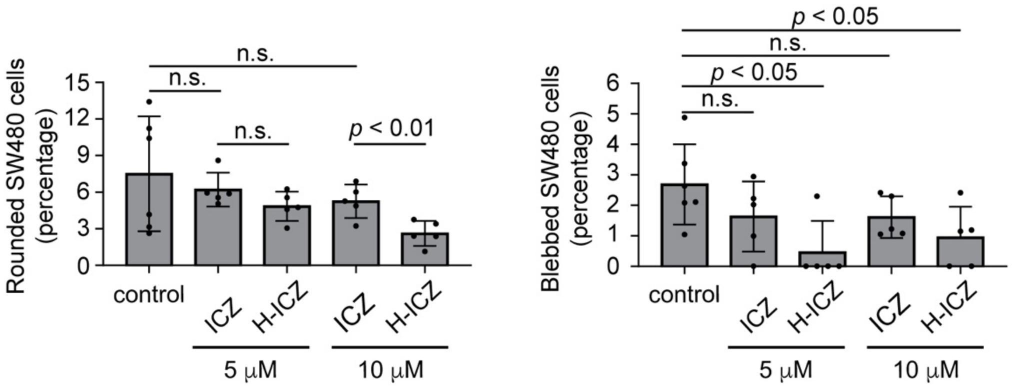

FIGURE S2 Effects of ICZ and H-ICZ on the morphology of SW480 cells. Non-metastatic SW480 cells were treated with DMSO solvent alone (control), 5 or 10 µM ICZ or H-ICZ for 5 hours prior to CD9 immunolabelling. Cell morphology was analyzed by CLSM. Bar graphs show the percentage of cells harboring a rounded morphology (left) or with plasma membrane blebbing (right) upon exposure to different concentrations of drugs. The mean ± S.D. are shown. At least 100 cells were evaluated per experiment and condition (n = 5-6). *P* values are indicated. N.s., not significant.

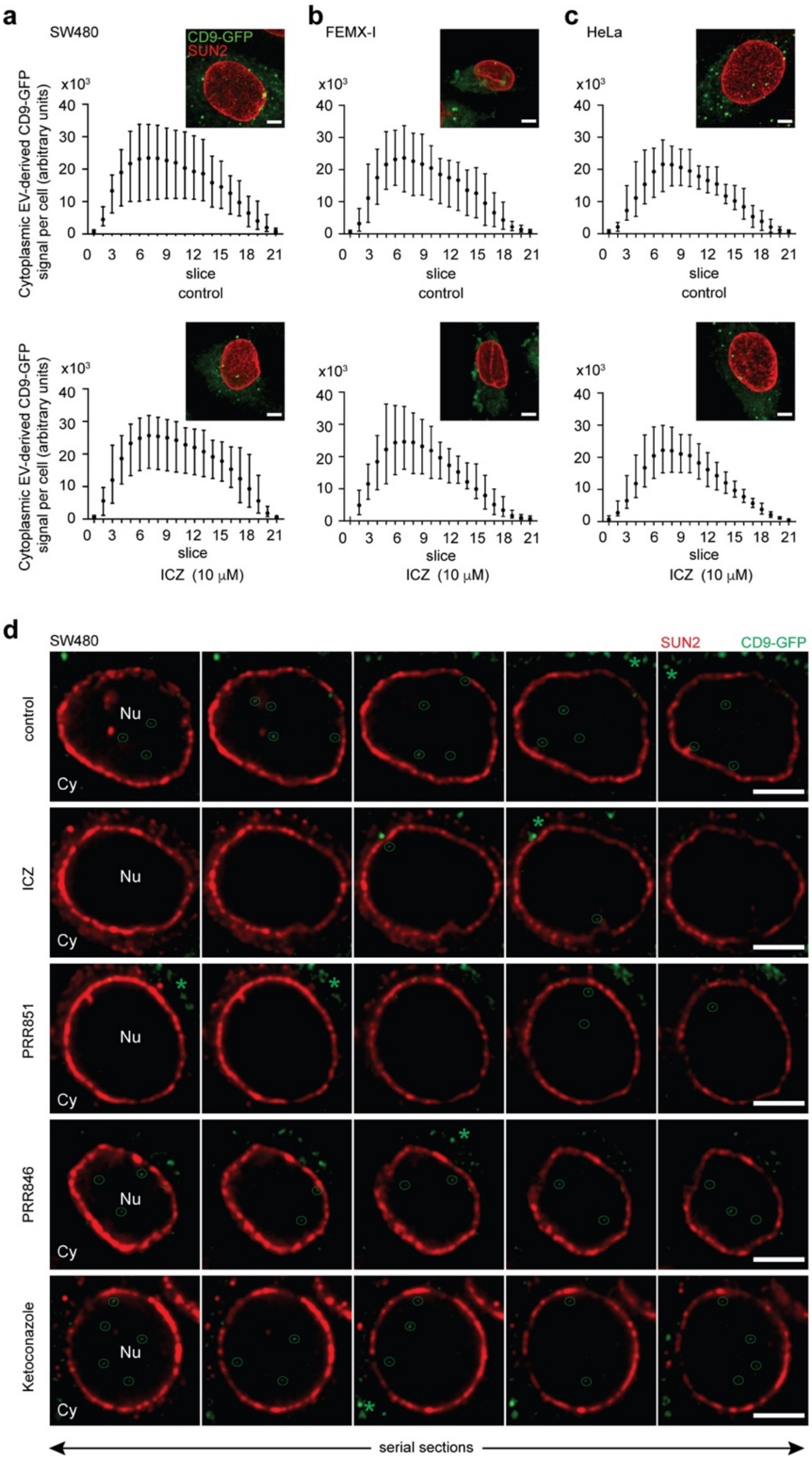

FIGURE S3 ICZ does not affect cell uptake of EV-associated CD9-GFP. (a-d) SW480 (a, d), FEMX-I (b) and HeLa (c) cells were pre-treated for 10 minutes with DMSO solvent alone (control) or with 10 µM ICZ, PRR851, PRR846 or ketoconazole prior to 5-hour incubation with FEMX-I cell-derived CD9-GFP^{+} EVs (1 x 10^{9} particles/ml) in the absence or presence of drugs. Recipient cells were then fixed and immunolabelled for SUN2. The cytoplasmic (a-c) and

nuclear (d) CD9-GFP signals were analyzed by CLSM and quantified using Fiji software. Means with the range of green fluorescence per slice from 10 individual representative cells are displayed as well as composites of 21 optical x-y sections (a-c, inset). A serial single x-y sections (0.2 µm each) of representative SW480 nuclei is shown (d). Individual CD9-GFP signals in the nucleus (Nu) are highlighted with green circles, while those in the cytoplasm (Cy) are indicated with green asterisks. Scale bars, 5 µm.

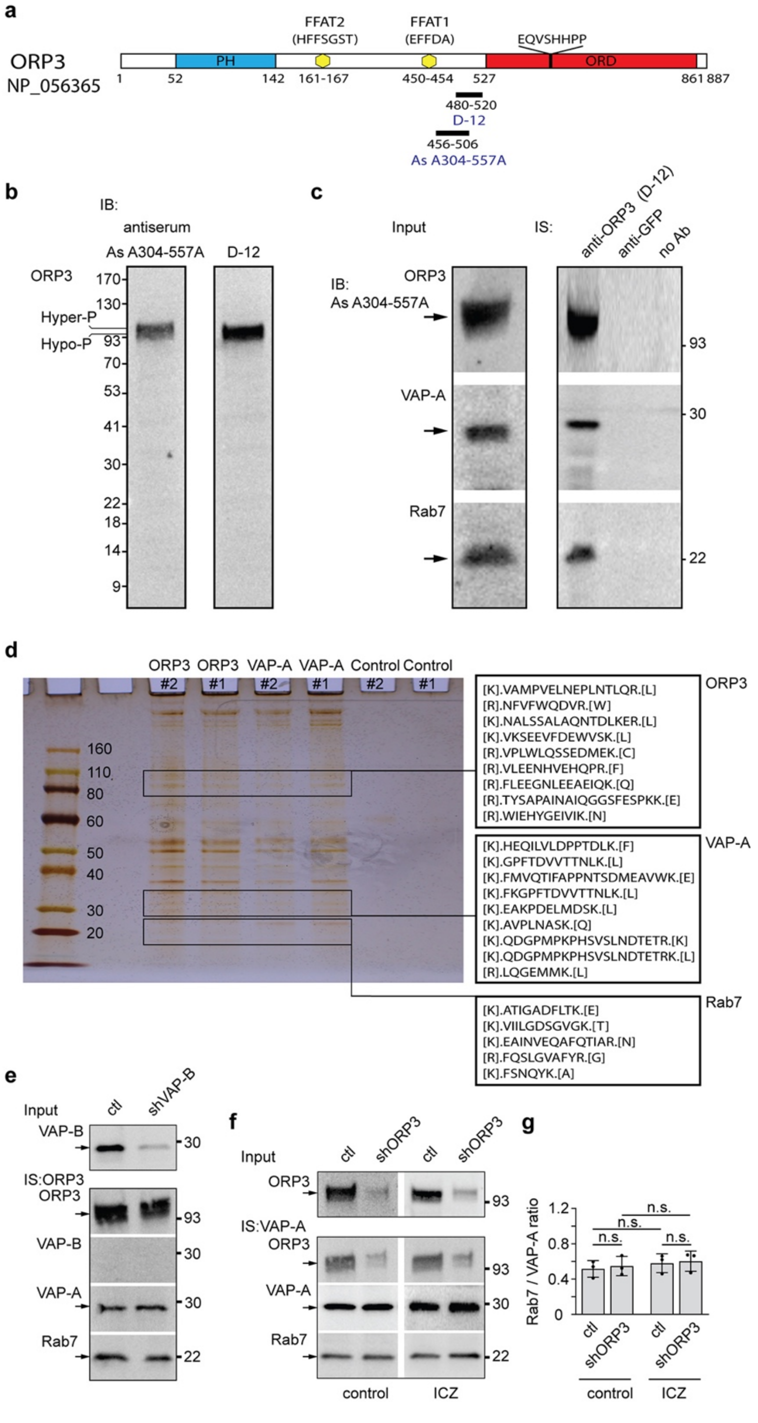

FIGURE S4 Characterization of anti-ORP3 antibodies and Co-immunoisolation of ORP3, VAP-A and Rab7. (a) Representation of the ORP3 protein (GenBank number NP_056365) and the specific localization of epitopes of monoclonal Ab D-12 and antiserum (AS) A304-557A. The location of distinct protein domains is indicated with numbers referring to amino acid residues. The N-terminal pleckstrin homology (PH) domain, the central two phenylalanine in

an acidic tract (FFAT) motifs, and the C-terminal OSBP-related domain (ORD) are indicated. The sequences of FFAT1 and FFAT2 motifs are shown in brackets. The highly conserved OSBP fingerprint signature sequence EQVSHHPP is marked in the ORD region. (b) Immunoblots (IB) of SW480 cell detergent lysates showing ORP3 immunoreactivities of the AS A304-557A and monoclonal Ab D-12. Note that ORP3 often appears as a close doublet, which corresponds to the hyper- and hypophosphorylated forms. (c) The cell lysates were subjected to immunoisolation (IS) using either monoclonal Ab D-12, or anti-GFP Ab, or without primary Ab as negative controls. Antibody-antigen complexes were recovered with Protein G-coupled magnetic beads. The input (1/50) and entire bound fractions were probed for ORP3 (using the As A304-557A), VAP-A, and Rab7. (d) Isolated proteins from either ORP3 or VAP-A-based IS were identified by mass spectrometry. As negative control, primary antibody was omitted. Two independent experiments (#1 and 2) were performed. Identified peptides of ORP3, VAP-A and Rab7 from both ORP3 or VAP-A IS are indicated. These are the sequence residues just before, and after, the trypsin cleavage sites (bracket). For comprehensive lists of peptides see Table S5. (e) Detergent lysates prepared from parental (scrambled control) and VAP-B-silenced (shVAP-B) SW480 cells were subjected to IS using anti-ORP3 Ab D-12 and Protein G-coupled magnetic beads. The input and bound fractions were probed by immunoblotting for ORP3 (AS A304-557A), VAP-B, VAP-A, and Rab7. See Figure 4e for the quantification of Rab7/ORP3 ratio. (f and g) Parental (ctl) and ORP3-silenced (shORP3) SW480 cells were incubated with either DMSO solvent alone (control) or 10 µM ICZ for 5 hours, solubilized and subjected to IS using anti-VAP-A Ab and Protein G-coupled magnetic beads. The input and bound fractions were probed for ORP3, VAP-A and Rab7 (f). The ratio of protein immunoreactivities of the indicated pairs was quantified (g, n = 3). The molecular mass markers are indicated, and arrows point to the proteins of interest. The mean ± S.D. are shown. N.s., not significant.

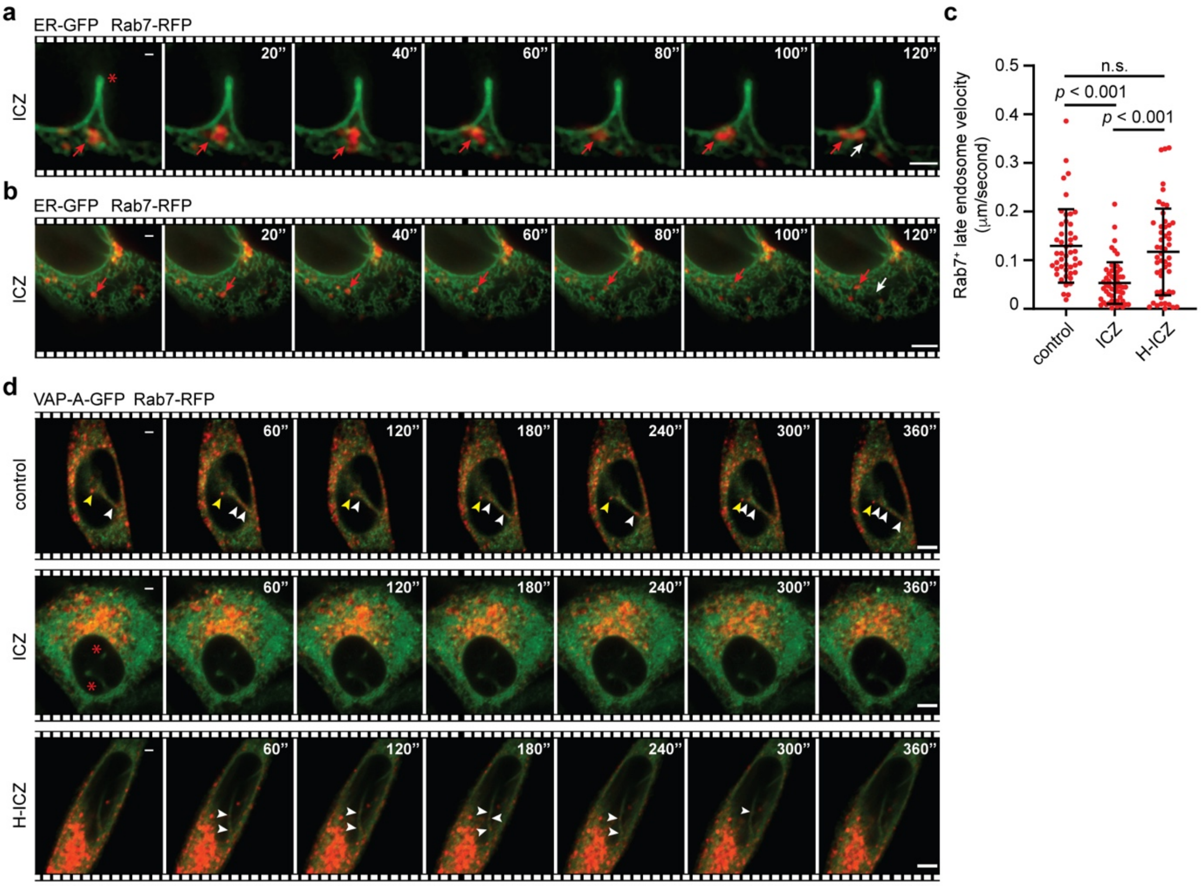

FIGURE S5 Trafficking of late endosomes into NEI of SW480 and FEMX-I cells. (a-d) SW480 cells co-expressing ER-GFP and Rab7-RFP (a-c) or FEMX-I cells co-expressing VAP-A-GFP and Rab7-RFP (d) were treated with DMSO solvent alone (control) or 10 μM drugs as indicated for 5 hours, and then recorded by time-lapse video microscopy every 20 seconds for a period of 2 minutes (a-c) or every 60 seconds for a period of 6 minutes (d). Elapsed time is indicated in the top right corner. Red arrows indicate Rab7-RFP^{+} late endosomes moving at the entry of NEI (a) or within cytoplasm (b). The velocity of the latter was quantified (c). The mean ± S.D. are shown (n ≥ 50 Rab7^{+} structures per condition). *P* values are indicated. N.s., not significant. White arrows point to the initial position of Rab7-RFP^{+} late endosomes. White arrowheads show Rab7-RFP^{+} late endosomes moving along VAP-A^{+} NEI, while yellow arrowheads indicate a Rab7-RFP^{+} late endosome that remained stationary for the duration of the video (d). Red asterisks indicate to VAP-A^{+} NEI without Rab7-RFP in the presence of ICZ. Scale bars, 5 μm.

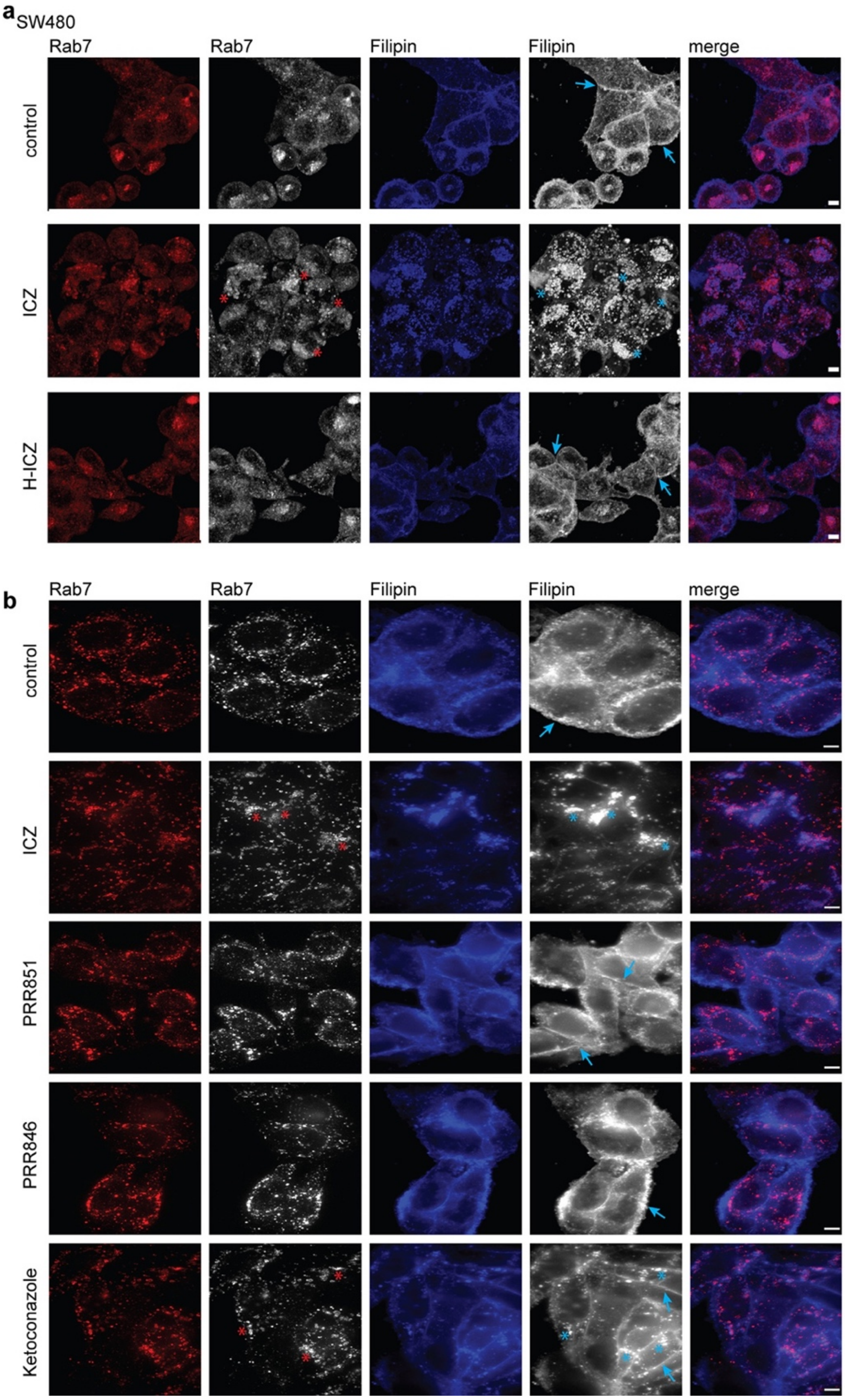

FIGURE S6 ICZ induces accumulation of cholesterol in Rab7^{+} late endosomes. (a, b) SW480 cells were incubated with DMSO solvent alone (control), 10 µM ICZ, H-ICZ, PRR851, PRR846 or ketoconazole for 5 hours prior to immunolabelling for Rab7 (red) and staining with filipin (blue). Cells were analyzed by CLSM. The corresponding black and white images are

also presented. Red and cyan asterisks indicate the cytoplasmic accumulation of Rab7 and cholesterol, respectively, upon ICZ or ketoconazole treatment. Arrows point to the presence of cholesterol in the plasma membrane in cells exposed to all conditions except ICZ. Scale bars, 5 μm.

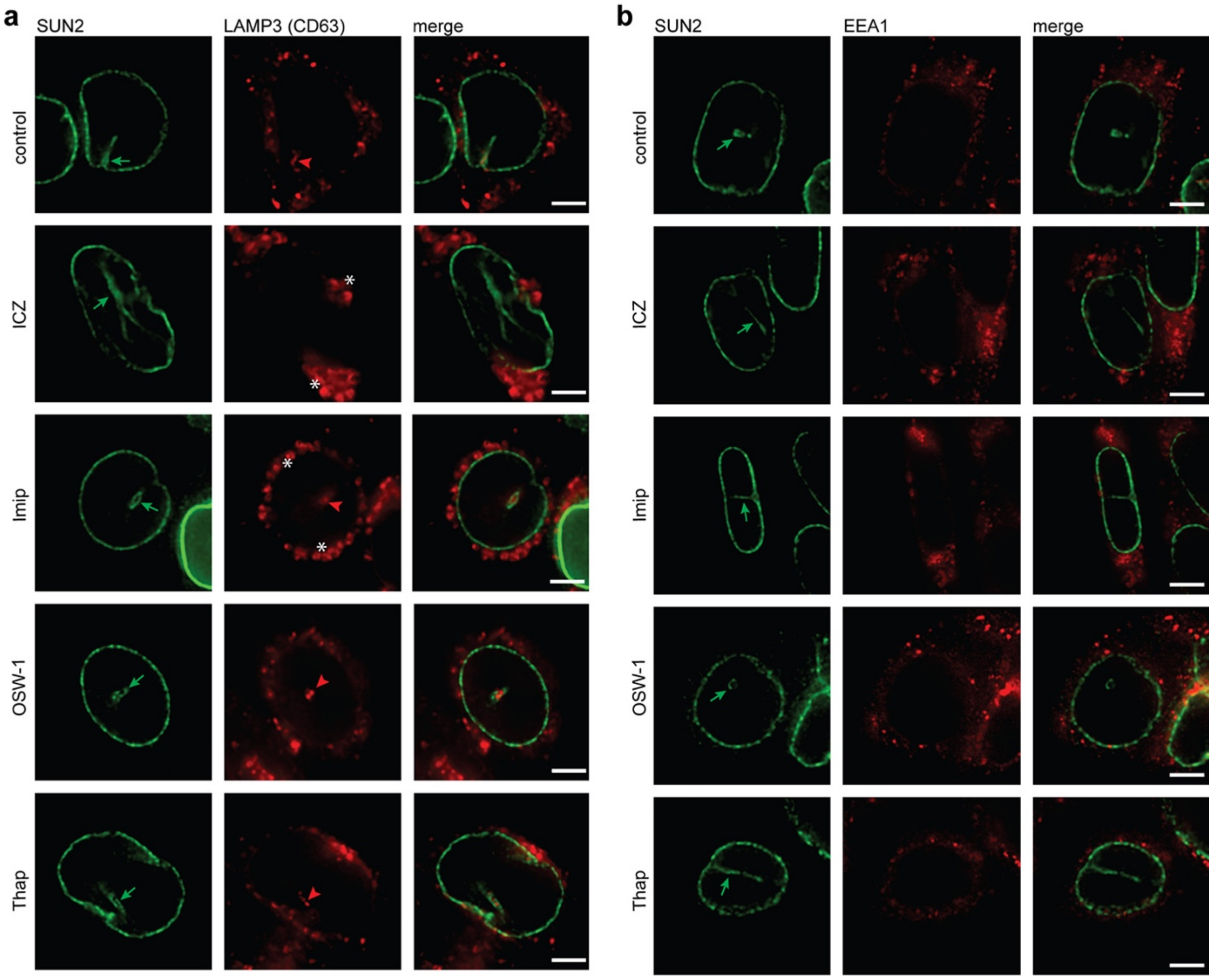

FIGURE S7 Presence of late endosome marker CD63 in the nucleoplasmic reticulum. SW480 cells treated for 5 hours with DMSO alone (control) or with 10 µM ICZ, 100 µM imipramine (Imip), 50 nM OSW-1 or 1 µM thapsigargin (Thap) prior to double immunolabelling for SUN2 and CD63 (a) or EEA1 (b). Cells were analyzed by CLSM. Green arrows point to NEI, while red arrowheads indicate CD63^{+} structures within NEI. Asterisks indicate the enlargement of CD63^{+} structures in ICZ- and imipramine-treated cells, which could correspond to the accumulation of cholesterol in late endosomes/lysosomes. Note the absence of EEA1 as a marker of early endosomes in NEI. Scale bars, 5 µm.

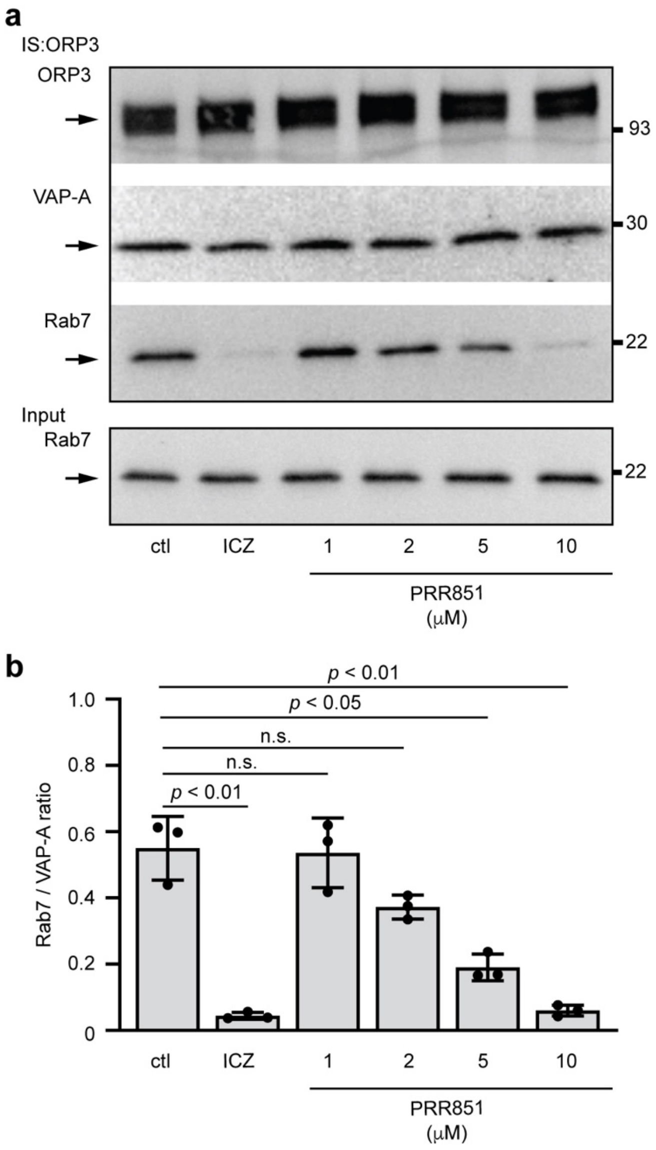

FIGURE S8 Effects of increasing concentration of PRR851 on the VOR complex. (a, b) SW480 cells were incubated with DMSO solvent alone (control), 10 μM ICZ or different PRR851 concentrations as indicated for 5 hours, solubilized and subjected to immunoisolation (IS) using anti-ORP3 Ab and Protein G-coupled magnetic beads. The input (1/50) and bound fractions were probed by immunoblotting for ORP3, VAP-A and Rab7 (a). The ratio of protein immunoreactivities of the indicated pairs was quantified (b, n = 3). Molecular mass markers are indicated, and arrows point to the proteins of interest. The mean ± S.D. are shown. *P* values are indicated. N.s., not significant.

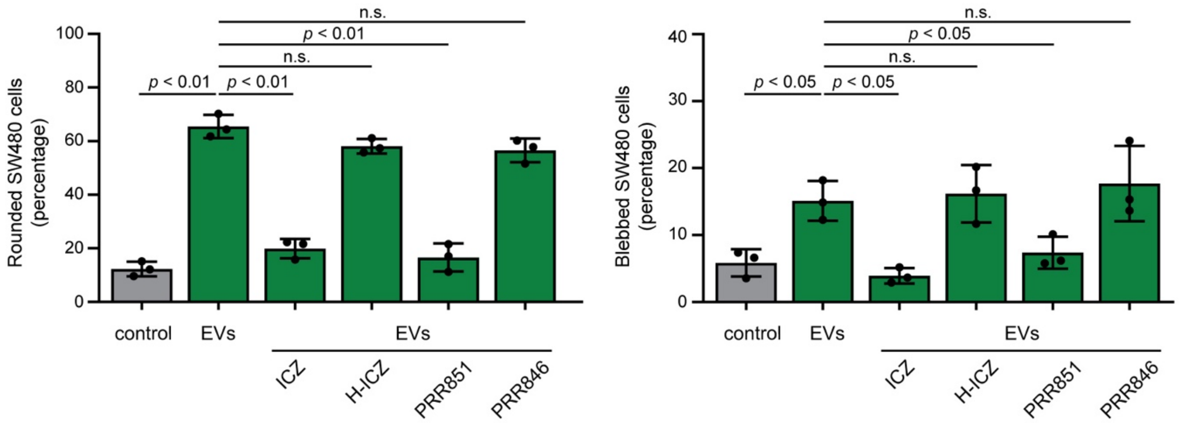

FIGURE S9 ICZ and PRR851 inhibit EV-induced pro-metastatic morphological transformation of SW480 cells. SW480 cells were pre-treated with DMSO (control), 10 µM ICZ, H-ICZ, PRR851 or PRR846 for 10 minutes prior to incubation without or with EVs (2 x 10^9 particles/ml) derived from SW620 cells for 5 hours prior to CD9 immunolabelling. Cell morphology was analyzed by CLSM. Bar graphs show the percentage of cells harboring a rounded (left) or blebbed (right) morphology upon exposure to EVs. The mean ± S.D. are shown. At least 100 cells were evaluated per condition and experiment (n = 3). *P* values are indicated. N.s., not significant.

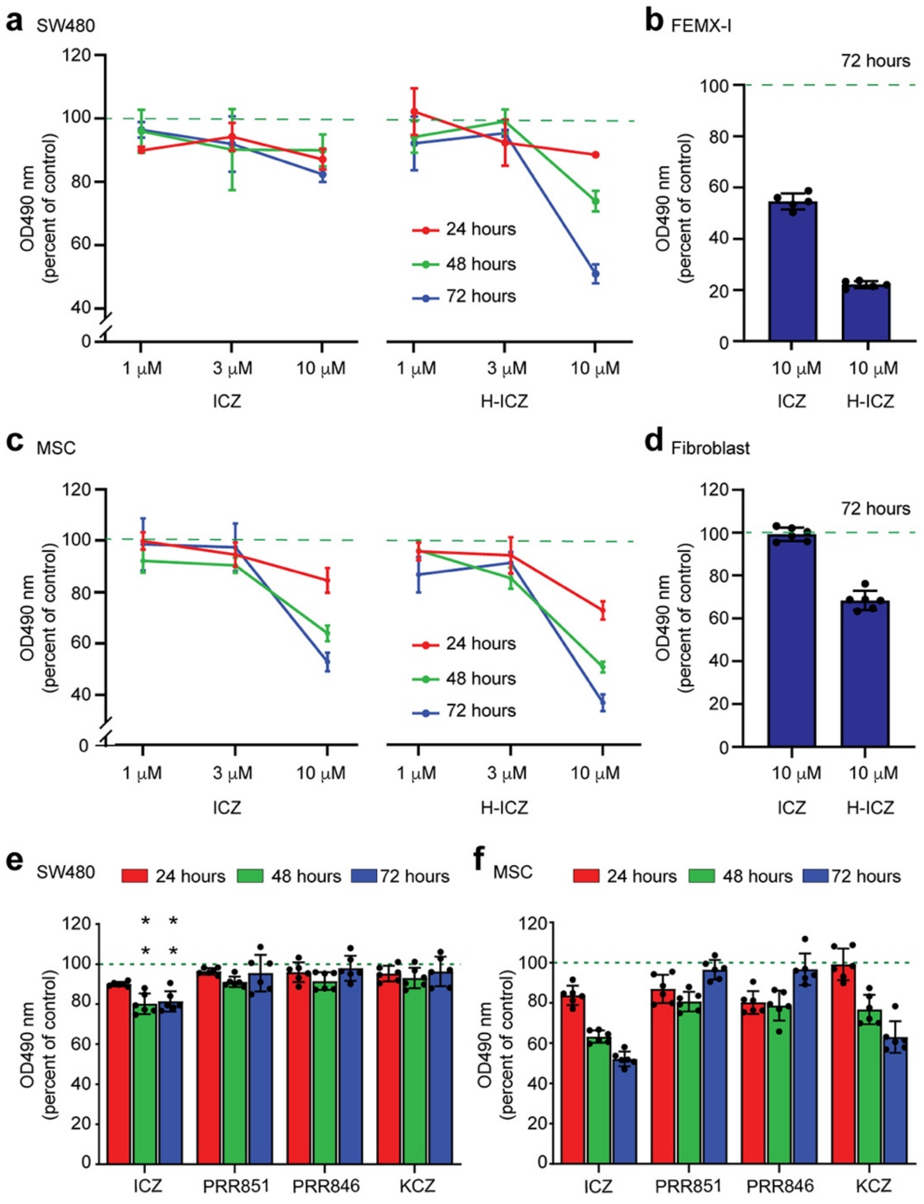

FIGURE S10 Cell growth inhibition effects of ICZ, PRR851 and PRR846. (a-f) Different cell lines as indicated were incubated with DMSO solvent alone (control), 1, 3 and 10 µM ICZ or H-ICZ (a-f), 10 µM PRR851, PRR846 or ketoconazole (KCZ) (e, f) for 24, 48 and 72 hours as indicated. At the end of each time point, MTS tetrazolium compound was added for 1 hour to evaluate the amounts of viable cells. The absorbance value was measured at 490 nm. The mean ± S.D. are shown (n = 6). **, $P < 0.01$.

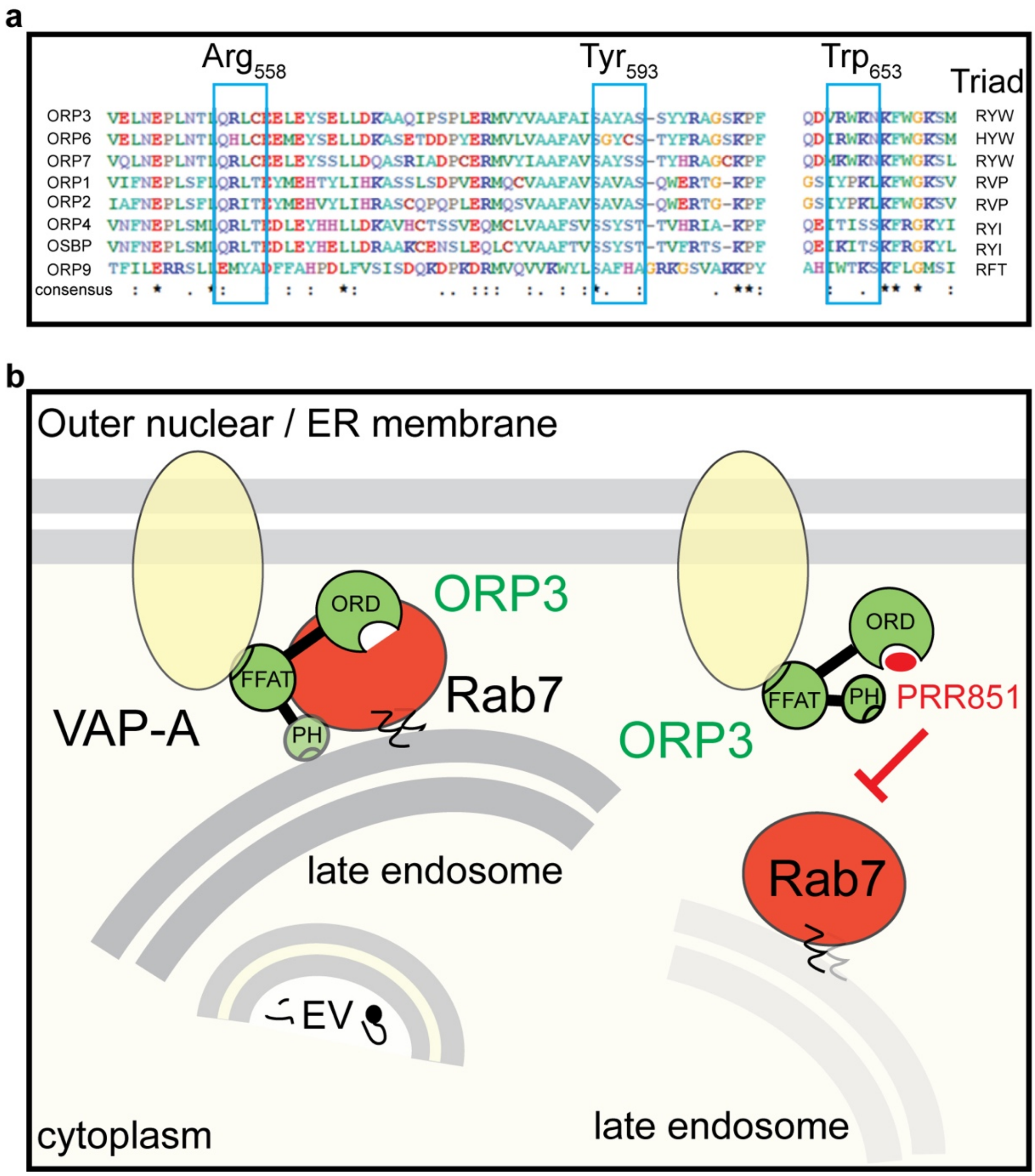

FIGURE S11 Potential model for the action of PRR851 on the VOR complex. (a) Multiple sequence alignment for seven ORP isoforms as indicated and human OSBP. Attention is focused on the region around the key residues (Arg558, Tyr594 and Trp653). The analysis reveals that only ORP7 retains the key triad (RYW), while all other analyzed sequences possess different amino acids maintaining at most two key residues. Thus, Arg558 (and the close Leu559) is the most conserved residue followed by Tyr593, while Trp653 is conserved only in 3 out of 8 aligned sequences. The amino acid residues are indicated with single code letters. (b) Inhibition of ORP3 by PRR851. Bridging the late endosomes containing the endocytosed EVs (EV) and

their cargo to the outer nuclear membrane involves the outer nuclear/ER membrane-associated VAP-A, ORP3 and late endosome-associated Rab7 proteins. The interaction between ORP3 and VAP-A occurs via two specific sequence motifs formed by two phenylalanines in an acidic tract (FFAT) in the ORP3 protein, while a pleckstrin homology (PH) domain mediates its interaction with phosphoinositides in non-ER organelles including late endosomes. The ORP3 domain engaged in the interaction with Rab7 remains to be defined, but its R-Ras-binding site could be involved. The interaction of PRR851 (red) with the C-terminal OSBP-related domain (ORD) of ORP3 (i.e. the hydrophobic pocket that binds to a single sterol) could lead to a conformational change of the protein that would interfere with its interaction with Rab7, and thus abrogate the integrity of the VOR complex.

Supplementary Video Legends

VIDEO S1 The video depicts SW480 cells co-expressing ER-GFP and Rab7-RFP treated with DMSO (control) for 5 hours, and then recorded by time-lapse video microscopy every 20 seconds for a period of 5 minutes. Still images from this movie are shown in Figure 5d (top panel). (Format avi; size 3.5 MB).

VIDEO S2 The video depicts SW480 cells co-expressing ER-GFP and Rab7-RFP treated with 10 µM H-ICZ for 5 hours, and then recorded by time-lapse video microscopy every 20 seconds for a period of 5 minutes. Still images from this movie are shown in Figure 5d (bottom panels). (Format avi; size 3.5 MB).

VIDEO S3 The video depicts SW480 cells co-expressing ER-GFP and Rab7-RFP treated with 10 µM ICZ for 5 hours, and then recorded by time-lapse video microscopy every 20 seconds for a period of 5 minutes. Still images from this movie are shown in Figure 5d (middle panels). (Format avi; size 3.5 MB).

VIDEO S4 The video depicts a high magnification of SW480 cells co-expressing ER-GFP and Rab7-RFP treated with 10 µM ICZ for 5 hours, and then recorded by time-lapse video microscopy every 20 seconds for a period of 5 minutes. Still images from this movie are shown in Figure S5a. Note that Rab7-RFP did not penetrate the NEI. (Format avi; size 4.9 MB).

VIDEO S5 The video depicts a high magnification of SW480 cells co-expressing ER-GFP and Rab7-RFP treated with 10 µM ICZ for 5 hours, and then recorded by time-lapse video microscopy every 20 seconds for a period of 5 minutes. Still images from this movie are shown

in Figure S5b. Note that Rab7-RFP is moving in the cytoplasmic area (Format avi; size 1.0 MB).

VIDEO S6 The video depicts FEMX-I cells co-expressing VAP-A-GFP and Rab7-RFP treated with DMSO (control) for 5 hours, and then recorded by time-lapse video microscopy every 60 seconds for a period of 10 minutes. Still images from this movie are shown in Figure S5d (top panel). (Format avi; size 13.4 MB).

VIDEO S7 The video depicts FEMX-I cells co-expressing VAP-A-GFP and Rab7-RFP treated with 10 μM ICZ for 5 hours, and then recorded by time-lapse video microscopy every 60 seconds for a period of 10 minutes. Still images from this movie are shown in Figure S5d (middle panel). (Format avi; size 13.4 MB).

VIDEO S8 The video depicts FEMX-I cells co-expressing VAP-A-GFP and Rab7-RFP treated with 10 μM H-ICZ for 5 hours, and then recorded by time-lapse video microscopy every 60 seconds for a period of 10 minutes. Still images from this movie are shown in Figure S5d (bottom panel). (Format avi; size 13.4 MB).

Supplementary Tables

TABLE S1 Primary antibodies used for immunodetection

Antigen	Clone/AS (host species)	Manufacturer	Dilution			Representative publications (PMID)	Validation in this study
			IS	IB	ICC		
Alix	3A9 (m)	Cell Signaling Technology		1:500		31731761	
Calnexin	AF18 (m)	Thermo Fisher Scientific		1:500		33441764	
CD9	P1/33/2 (m)	Santa Cruz Biotechnology		1:500	1:50	25103498	
CD63	Ts63 (m)	Thermo Fisher Scientific		1:500		29539618	
CD63 (LAMP3)	MEM-259 (m)	Thermo Fisher Scientific			1:100	28674187	
CD81	D-4 (m)	Santa Cruz Biotechnology		1:500		30639512	
EEA1	AS PA1-063A (r)	Thermo Fisher Scientific			1:50	28179995	
GFP	B-2 (m)	Santa Cruz Biotechnology	1:200			31515488	
Histone H1	AE-4 (m)	Santa Cruz Biotechnology		1:500		31362387	
Rab7	EPR7589 (r)	Abcam		1:500	1:50	31085713	
SUN2	A-10 (m)	Santa Cruz Biotechnology			1:50	30982221	
ORP3	D-12 (m)	Santa Cruz Biotechnology	1:200	1:500		30018135	Fig. S4b,c
	AS A304-557A (r)	Bethyl Laboratories		1:1000	1:50		Fig. S1a,b Fig. S4b,c,f
VAP-A	4C12 (m)	Novus Biologicals	1:500		1:100	30540949	Fig. S1a,b
	AS A304-366A (r)	Bethyl Laboratories		1:1000			Fig. 4d
VAP-B	AS A302-894A (r)	Bethyl Laboratories			1:50	26957043	Fig. S1a,b Fig. S4e

AS, antiserum; M, mouse; R, rabbit; IS, immunoisolation; IB, immunoblotting; ICC, immunocytochemistry

TABLE S2 Secondary antibodies used for immunodetection

Antibody	Fluorophore	Manufacturer	Dilution	
			IB	ICC
Donkey anti-mouse IgG	FITC	Jackson ImmunoResearch Laboratories	1:500	1:50
Donkey anti-rabbit IgG	FITC	Jackson ImmunoResearch Laboratories	1:500	1:50
Donkey anti-rabbit IgG	TRITC	Jackson ImmunoResearch Laboratories		1:50
Goat anti-mouse IgG	Atto 488	Rockland Immunochemicals		1:1000
Goat anti-rabbit IgG	Alexa Fluor 647	Thermo Fisher Scientific		1:2000

FITC, fluorescein isothiocyanate; TRITC, tetramethylrhodamine; IB, immunoblotting; ICC, immunocytochemistry

TABLE S3 Cell lines used in this study

Cell Lines	Origin	Source	Cell Media†	Validation (PMID)
SW480	Dukes' type B, colorectal adenocarcinoma	ATCC CCL-228	RPMI	28680152
SW620	Dukes' type C, colorectal adenocarcinoma	ATCC CCL-227	RPMI	28680152
FEMX-I	Melanoma	Lymph node from pt. with malignant melanoma	RPMI	30018135
HeLa	Cervical adenocarcinoma	ATCC CCL-2	DMEM	31790455
BJ	Normal fibroblast	ATCC CRL-2522	DMEM	27179759
Primary MSC		Bone marrow	MEMα	28129640

†All cell media were supplemented with 10% fetal bovine serum, 2 mM L-glutamine, 100 U/mL penicillin and 100 µg/mL streptomycin

Abbr. ATCC, American Type Culture Collection; pt., patient; RPMI, Roswell Park Memorial Institute 1640 Medium; DMEM, Dulbecco's Modified Eagle Medium; MEMα, Minimum Essential Medium alpha; PMID, PubMed Identification

TABLE S4 Quantification of EV-derived CD9-GFP in the nucleoplasm of host cells

Cell line	SW480				FEMX-I				HeLa			
Experiments	#1	#2	#3	Mean ± S.D.	#1	#2	#3	Mean ± S.D.	#1	#2	#3	Mean ± S.D.
Control	20.66[1]	17.76	19.16	19.19 ± 1.45	20.96	16.88	19.88	19.24 ± 2.11	14.14	16.44	15.08	15.22 ± 1.16
2 µM ICZ	14.34	10.48	14.6	13.14 ± 2.31	12.08	14.08	11.96	12.71 ± 1.19	8.42	9.84	10.44	9.57 ± 1.04
10 µM ICZ	4.94	3.52	4.84	4.43 ± 0.79	4.86	4.48	3.72	4.35 ± 0.58	5.52	4.76	3.84	4.71 ± 0.84

[1]Number of individually punctate GFP signal per nucleus. 50 cells were analyzed per condition.

TABLE S5 VAP-A, ORP3 and Rab7 are co-immunoisolated using anti-VAP-A or ORP3 antibodies

An Excel workbook that summarizes evidence for the presence and relative abundance of three proteins identified (VAP-A, ORP3 and Rab7) is presented in a separate file (Table S5.xlsx). For more details, see also Supplementary Materials and Methods (Mass spectrometry).
